# Ion‐Pair Interactions in Polyphenylene‐Based Quaternized Membranes Designed for Phosphoric Acid‐Doped Proton Exchange Membranes for High‐Temperature Fuel Cells

**DOI:** 10.1002/advs.202509467

**Published:** 2025-07-03

**Authors:** Lin Guo, Kenji Miyatake, Ahmed Mohamed Ahmed Mahmoud, Fanghua Liu, Fang Xian, Vikrant Yadav, Xiaofeng Hao, Shuanjin Wang, Yuezhong Meng

**Affiliations:** ^1^ Clean Energy Research Center University of Yamanashi Kofu Yamanashi 400–8510 Japan; ^2^ Hydrogen and Fuel Cell Nanomaterials Center University of Yamanashi Kofu Yamanashi 400–8510 Japan; ^3^ Department of Applied Chemistry Waseda University Tokyo 169–8555 Japan; ^4^ School of Materials Science and Engineering Sun Yat‐sen University Guangzhou 510275 China; ^5^ Institute of Chemistry Henan Provincial Academy of Science Zhengzhou 450000 China

**Keywords:** high‐temperature fuel cells, ion‐pair interaction, phosphoric acid doping, polyphenylene, proton exchange membranes

## Abstract

Phosphoric acid (PA)‐doped proton exchange membranes (PEMs) face significant challenges owing to the loss of PA, particularly under high humidity conditions. Ion‐pair interactions between PA and quaternary ammonium (QA) groups can effectively mitigate PA loss. Herein, polyphenylene‐based quaternized membranes (BAF‐QAF and C7‐QAF) comprising distinct hydrophobic moieties [BAF = (perfluoropropane‐2,2‐diyl)dibenzene and C7 = 1,1‐diphenylcycloheptane] and fluorenyl groups with pendant QA head groups are designed and used as PA‐doped PEMs with low or no fluorine contents to realize high‐temperature and low‐humidity operability. The resulting membranes exhibited excellent PA retention, maintaining >85% of their initial proton conductivities at 90% relative humidity after 10 humidity cycles. PA‐doped membranes PA‐C7‐QAF and PA‐BAF‐QAF exhibit superior proton conductivities of 60.3 and 58.4 mS cm^−1^ at 160 °C, respectively. PA‐C7‐QAF and PA‐BAF‐QAF fuel cells deliver peak power densities of 0.579 and 0.537 W cm^−2^ at 140 °C and 0.706 and 0.640 W cm^−2^ at 160 °C, respectively, under dehumidified conditions. The PA‐C7‐QAF cell also exhibits impressive durability with an average voltage decay of 30 µV h^−1^ (140 °C, 0.15 A cm^−2^) after an initial voltage drop. These findings underscore PA‐C7‐QAF and PA‐BAF‐QAF membranes as promising components in high‐temperature fuel cells.

## Graphical abstract

Quaternized polyphenylene‐based membranes with tailored hydrophobic groups (C7‐QAF and BAF‐QAF) exhibit high phosphoric acid uptake, acid retention, proton conductivity, and stability. Their well‐developed microphase‐separated structures and robust ion‐pair interactions enable superior fuel cell performance at 140 and 160 °C without humidification, highlighting their promise as low‐fluorine/fluorine‐free proton exchange membranes for high‐temperature fuel cells.

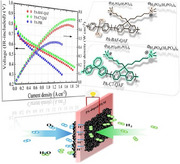

## 1 Introduction

Proton exchange membrane fuel cells (PEMFCs) comprising perfluorosulfonic acid (PFSA) membranes such as Nafion have been commercialized in electric vehicles and residential cogeneration systems. PEMFCs generally operate at 80 °C or less under hydrated conditions owing to material restrictions. The operation of PEMFCs at high temperatures (>120 °C) will reduce the system cost by lowering cooling and humidification requirements while improving their electrocatalyst activity and tolerance to contaminants [e.g., carbon monoxide (CO) or hydrogen sulfide (H_2_S)] present in reformed hydrogen.^[^ ^1^, ^2^ ^]^ The use of phosphoric acid (PA)‐doped polybenzimidazole (PBI) membranes in high temperature–operated PEMFCs (120–180 °C) without external humidification and/or extensive heat management was investigated. However, PA‐doped PBI membranes suffer from PA leaching, especially when in contact with water during start‐up and shut down when water vapor is condensed.^[^ ^3^, ^4^ ^]^ PA leaching by water occurs owing to the weak acid–base interaction of PA with the imidazole groups of PBI. The binding energy of PA and imidazole units is low (≈17.4 kcal mmol^−1^).^[^ ^5^, ^6^ ^]^

To mitigate PA leaching from the membranes, enhancing the interaction between PA and the polymer matrix is essential. An effective strategy is the coordination of PA with quaternary ammonium (QA) groups. Kim et al. demonstrated that the energy of the ion‐pair interaction between the benzyltrimethylammonium group and biphosphate anion in the PA‐doped polymer (QAPOH) membrane was 151.7 kcal mol⁻^1^, which was >8‐fold stronger than that of the acid–base interaction in the PA‐PBI system.^[^ ^6^ ^]^ This significantly strong interaction effectively mitigated PA from leaching the membrane. The PA‐doped QAPOH membrane with an acid doping level (ADL, the number of PA molecules per PA‐absorbing site) of ≈14.4 lost 40% of adsorbed PA after five humidity cycles [5–90% relative humidity (RH) at 80 °C]. In contrast, the PA‐doped PBI membrane (ADL = ≈11.6) lost 68% of adsorbed PA after a single humidity cycle under the same conditions. Both membranes exhibited a proton conductivity of >10 mS cm^−1^ at 5% RH in the first humidity cycle. The conductivity of the PA‐doped PBI membrane significantly decreased to <0.1 mS cm^−1^ in the second cycle, whereas that of the PA‐doped QAPOH membrane gradually decreased and stabilized at >0.1 mS cm^−1^ after the fifth humidity cycle. The PA‐doped QAPOH membrane exhibited superior hydrogen (H_2_)/oxygen (O_2_) fuel cell performance, achieving a peak power density of >0.700 W cm^−2^, while the commercial PA‐doped PBI (Celtec) membrane exhibited a power density of <0.700 W cm^−2^ under identical conditions (160 °C and 8% RH). Furthermore, the PA‐doped QAPOH membrane exhibited durability for 500 h at 120 °C (H_2_/air) without performance degradation caused by PA loss and/or PEM degradation. Bae et al. investigated a series of PA‐doped ion‐pair coordinated PEMs (PA‐BPN1‐TMA, PA‐BPN1‐Pip, and PA‐BPN1‐DMIm) featuring three QA groups: trimethylalkylammonium (TMA), 1‐methylpiperidium (Pip), and 1,2‐dimethylimidazolium (DMIm).^[^ ^7^ ^]^ PA‐BPN1‐TMA (ADL ≈7.4), PA‐BPN1‐Pip (ADL ≈6.0), and PA‐BPN1‐DMIm (ADL ≈7.7) underwent four successive humidity cycles (5–90% RH at 80 °C). The three membranes exhibited slightly higher conductivity in the first humidity cycle than the subsequent cycles, which was attributed to the minor loss of free PA from the membrane surface. The comparable conductivity values of PA‐BPN1‐TMA, PA‐BPN1‐Pip, and PA‐BPN1‐DMIm in the range of 5–90% RH were ≈30–300 mS cm^−1^, 15–250 mS cm^−1^, and 15–300 mS cm^−1^, respectively. These three PEMs exhibited high tolerance of proton conductivity to humidity at 80 °C. No significant conductivity changes were observed at 70% RH, while they retained ≥88% and ≥67% of their initial conductivity at 80% and 90% RH, respectively. Zhang et al. developed quaternized poly(fluorene alkylene‐co‐biphenyl alkylene) membranes with triply quaternized ammonium (tQA) side chains (PFBA‐tQA) for PA doping.^[^ ^8^ ^]^ The PA‐doped PFBA‐tQA membrane (ADL = 5.65) retained 94.6% of PA after ≈50 h at 80 °C and 40% RH. During humidity cycling tests at 80 °C, conductivity decreased slightly in the first two cycles, followed by stabilization in the third cycle with the proton conductivity ranging from 4 to 60 mS cm^−1^ at 10–90% RH. In H_2_/O_2_ fuel cell operation at 160 °C under dehumidified conditions, the membrane achieved a peak power density of 0.560 W cm^−2^. It demonstrated stable performance during the durability test at 160 °C and 0% RH, with an average voltage decay of 13 µV h^−1^ over 300 h at a constant current density of 0.2 A cm^−2^. Chen et al. prepared a hyperbranched polybenzyl crosslinked PBI membrane containing QA hydroxides (QOPBI‐15) and compared it with a poly(aryl ether benzimidazole) (OPBI) membrane.^[^ ^9^ ^]^ QOPBI‐15 with a PA uptake of ≈166.7% exhibited a proton conductivity of 49 mS cm^−1^ at 160 °C and 0% RH, which was 14 mS cm^−1^ higher than that of the PA‐doped OPBI membrane (35 mS cm^−1^) even though the PA‐doped OPBI membrane exhibited a higher PA uptake of ≈207.4%. The superior conductivity of the PA‐doped QOPBI‐15 membrane was attributed to its higher H_2_PO_4_⁻ density owing to QA groups in the hyperbranched crosslinker, which provided additional proton transfer sites. Benefited from the strong ionic interactions of biphosphate–ammonium ion pairs, PA‐doped QOPBI‐15 retained 68.5% of PA after 96 h at 80 °C and 40% RH, whereas PA‐doped OPBI retained only 60.0% of PA under the same conditions. In H_2_/O_2_ fuel cell tests at 160 °C and 0% RH, PA‐doped QOPBI‐15 achieved a peak power density of 0.260 W cm^−2^, which was 0.070 W cm^−2^ higher than that of PA‐doped OPBI (0.190 W cm^−2^). These results underscore the importance of strong ion‐pair interactions between PA and QA groups for achieving high and stable proton conductivity and the superior high‐temperature fuel cell (HT‐FC) performance of the resulting PA‐doped PEMs.

We earlier reported an anion conductive polymer quaternized poly(arylene perfluoroalkylene) (QPAF‐4) membrane containing perfluoroalkylene and fluorenyl groups with pendant QA groups for alkaline fuel cells and water electrolyzers.^[^ ^10^ ^]^ When doped with PA, QPAF‐4 membranes could also function as high‐temperature PEMs.^[^ ^11^ ^]^ The PA‐doped QPAF‐4 membrane (QPAF‐4‐150%PA; a PA uptake of ≈150% and ADL of ≈7.2) demonstrated a proton conductivity of 52 mS cm^−1^ at 160 °C and 0% RH. This value surpassed the conductivity of the PBI‐based membrane (48 mS cm^−1^, PA uptake = 230%) despite its lower PA uptake. In H_2_/O_2_ fuel cell tests, QPAF‐4‐150%PA achieved a peak power density of 0.683 W cm^−2^ at 160 °C under dry conditions, which is ≈ 0.120 W cm^−2^ higher than that of the PA‐doped PFBA‐tQA membrane. The cell was stable for 60 h with an average voltage decay of 200 µV h^−1^ at 140 °C and 0% RH at 0.15 A cm^−2^. This high performance was attributed to the strong PA–QA interactions and microphase‐separated morphology.

Based on these results, we designed and synthesized polyphenylene‐based quaternized membranes [(perfluoropropane‐2,2‐diyl)dibenzene (BAF)‐QAF and 1,1‐diphenylcycloheptane (C7)‐QAF] with reduced or zero fluorine contents and used them as PA‐doped PEMs for high‐temperature and low‐humidity operation. The polyphenylene backbone was chosen to significantly enhance microphase separation. In fact, polyphenylene ionomer (SPP‐QP)‐incorporated PBI composite membrane [PBI/SPP‐QP(10)] achieved an outstanding proton conductivity (>300 mS cm^−1^ at 160 °C, 0% RH) and a peak power density of 0.719 W cm^−2^ (160 °C, 0% RH, H_2_/O_2_) in a fuel cell.^[^ ^12^ ^]^ Herein, polyphenylene with fluorenyl groups bearing pendant QA groups was designed, which exhibited good membrane‐forming capabilities and mechanical stretchability. BAF‐QAF and C7‐QAF membranes comprising different hydrophobic moieties (BAF and C7) were expected to exhibit high proton conductivity, PA retention, and fuel cell performance. In particular, the C7 unit was expected to disrupt tight chain packing and promote more pronounced microphase separation compared to six‐membered rings, leading to better proton transport and PA retention. The effects of different hydrophobic moieties on PA uptake, mechanical properties, micromorphology, proton conductivity, PA retention, and fuel cell performance of the membrane were systematically studied.

## 2 Results and Discussion

### 2.1 Polymer Synthesis

**Scheme 1:**
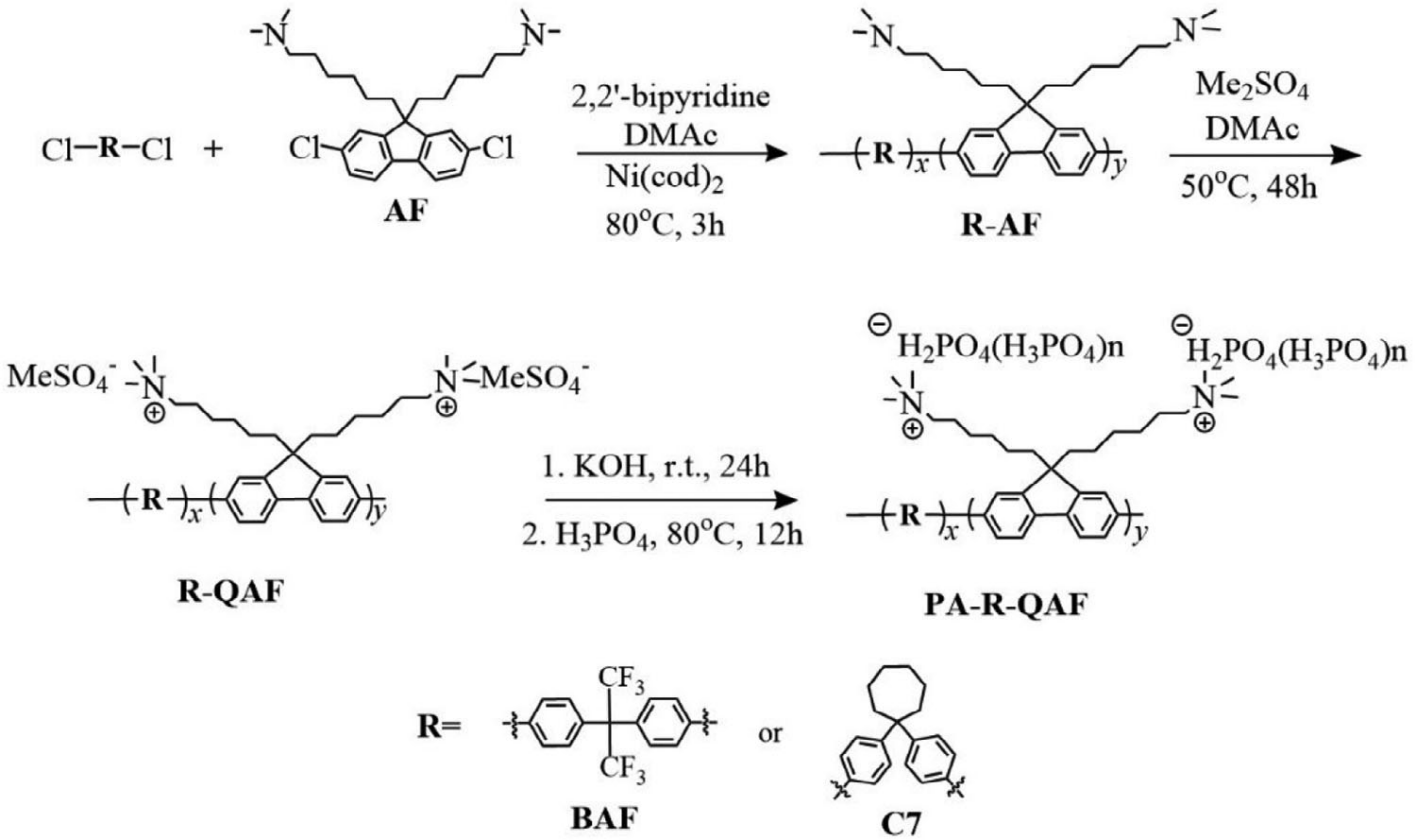
Synthesis of BAF‐AF, C7‐AF, BAF‐QAF, and C7‐QAF copolymers.

**Figure 1:**
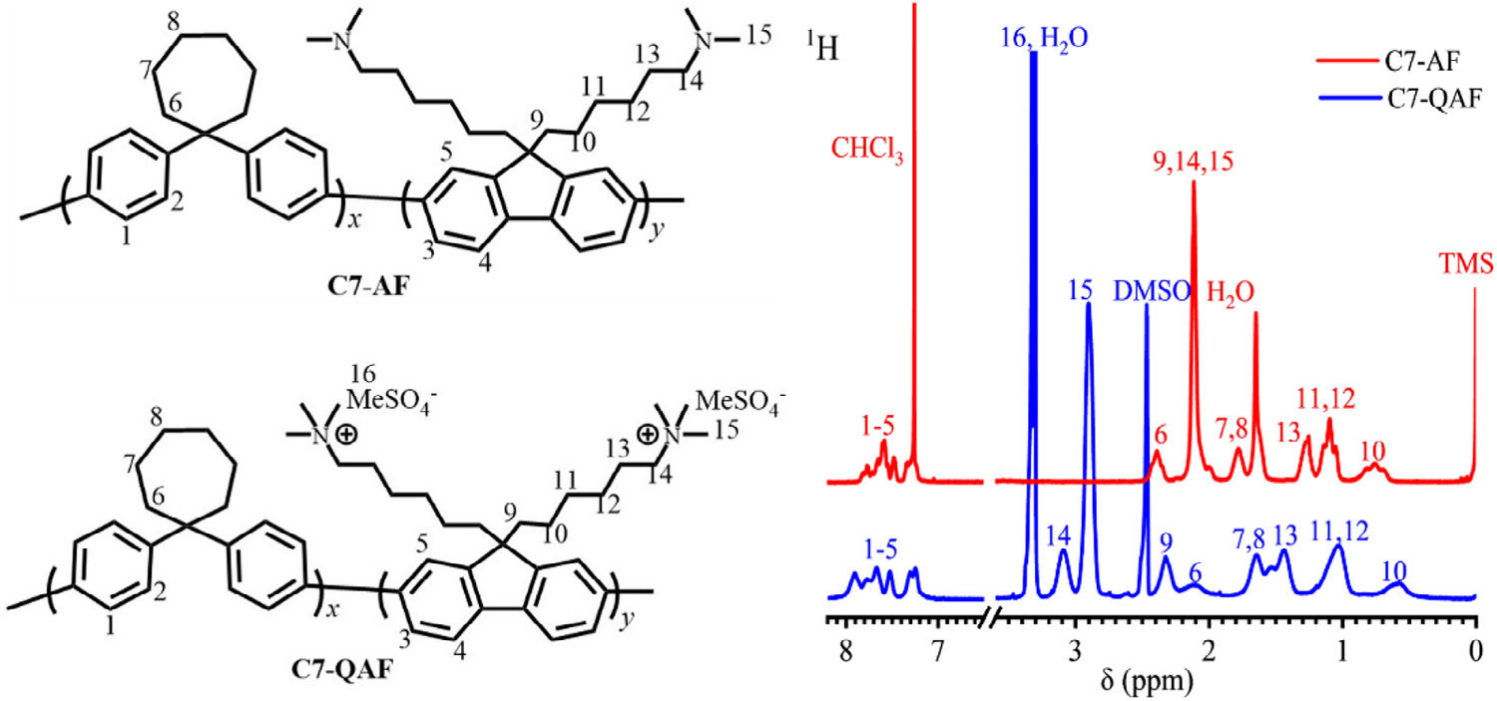
Structures and ^1^H NMR spectra of C7‐AF and C7‐QAF in CDCl_3_ and DMSO‐*_d6_* at room temperature.

Two copolymers (BAF‐AF and C7‐AF) comprising different hydrophobic (BAF and C7) but identical hydrophilic (AF) components were synthesized and quaternized, as shown in **Scheme** **1**. Using a nickel (Ni)(0)‐mediated polycondensation reaction, BAF‐AF and C7‐AF polymers were obtained in high yields (>97%) with reasonably high molecular weight (*M*_w_) and number average molecular weight (*M*_n_) values: BAF‐AF: *M*_w_ = 86.10 kDa, *M*_n_ = 21.22 kDa, and *M*_w_/*M*_n_ = 4.06; C7‐AF: *M*_w_ = 86.89 kDa, *M*_n_ = 13.15 kDa, and *M*_w_/*M*_n_ = 6.60. The *M*_w_ values of BAF‐AF and C7‐AF copolymers are comparable, indicating that the reactivities of BAF and C7 monomers are similar. The hydrophobic to hydrophilic monomer ratios in BAF‐AF and C7‐AF were 0.88:1.0 and 0.72:1.0, respectively, to ensure a high density of the ammonium groups (>3 mmol g⁻¹) and improve the polymer solubility. BAF‐AF and C7‐AF are soluble in common organic solvents such as chloroform, N,N‐dimethylacetamide (DMAc), and *N,N*‐dimethylformamide (DMF) but insoluble in dimethyl sulfoxide (DMSO). The pendant alkyl chains in the AF monomer are responsible for the good solubility of the copolymers. The proton nuclear magnetic resonance (^1^H NMR) spectrum of C7‐AF is presented in **Figure** **1**, confirming the structure of the copolymer. The signals appearing in the range of 7.2–8.0 are attributed to aromatic groups, while those in 0.4–3.4 ppm are attributed to alkylene (on AF) and cycloheptyl (on C7) groups.

The quaternization reaction was conducted using dimethyl sulfate in DMAc under mild conditions (50 °C for 48 h). BAF‐QAF and C7‐QAF were obtained in high yields (>95%) and were soluble in polar organic solvents (such as DMAc, DMF, and DMSO) but insoluble in nonpolar solvents (such as chloroform) owing to the strong hydrophilic QA groups. The structure of the quaternized copolymer (C7‐QAF) analyzed via ^1^H NMR (Figure 1) exhibits a new signal (Signal 16) at 3.37 ppm, as compared to C7‐AF, which is assigned to methyl sulfate (MeSO_4_^−^) groups present as counter anions. The signals of methyl groups attached to nitrogen atoms (Signal 15) shift downfield from 2.1 ppm (C7‐AF) to 2.9 ppm after the quaternization reaction, indicating the successful quaternization of C7‐AF. The IEC values of BAF‐QAF and C7‐QAF calculated from the signal integrals in the ^1^H NMR spectra are 3.12 and 3.03 mmol g^−1^, respectively, which are in good agreement with the target IEC (3.0 mmol g^−1^) calculated from the copolymer composition assuming a complete quaternization reaction. The copolymers were fabricated into flexible and transparent membranes via a solution‐casting method using DMAc as the solvent, as shown in Figure S4a,b (Supporting Information). Before PA doping, the copolymer membranes were treated with 1 M of potassium hydroxide (KOH) to convert the ammonium groups into their hydroxide ions (OH^−^).

### 2.2 Thermal Stability of Membranes

**Figure 2:**
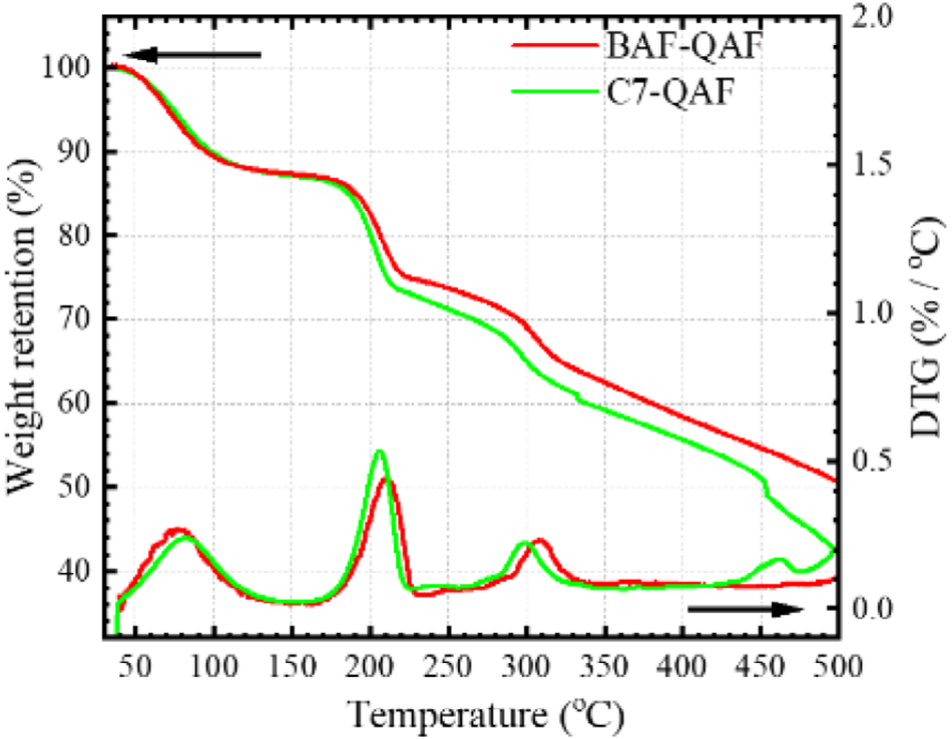
TGA curves of BAF‐QAF and C7‐QAF membranes.

The as‐prepared BAF‐QAF and C7‐QAF membranes (both in OH^−^ form) were subjected to thermogravimetric analysis (TGA), as shown in **Figure** **2**. Both membranes exhibit a ≈12% weight loss from 30 to ≈100 °C, which is attributed to dehydration. The second weight loss starting at ≈180 °C might be owing to the decomposition of ammonium groups.^[^ ^13^ ^]^ The second weight loss (≈13% for BAF‐QAF and ≈12% for C7‐QAF) is in agreement with the weight percentage of the QA cations (11% for both copolymers). The third weight loss begins at ≈220 °C and continues until the end of the measurement range (500 °C). The weight loss (≈26% for BAF‐QAF and 30% for C7‐QAF) nearly corresponds to the weight fraction of hexyl groups (25% for both copolymers) connecting QA groups to the polymer backbone. The TGA curves of PA‐doped membranes (PA‐BAF‐QAF and PA‐C7‐QAF) are presented in Figure S3 (Supporting Information) and compared with those of the pristine membranes (BAF‐QAF and C7‐QAF). The decomposition of the ammonium groups in the PA‐doped membranes occurred at a slightly lower temperature (≈170 °C) than in the pristine membranes (≈180 °C). However, the decomposition proceeded at a much lower degree in the PA‐doped membranes and was completed at ≈440 °C. By the end of the measurement range (500 °C), the total weight losses of the PA‐BAF‐QAF and PA‐C7‐QAF membranes were ≈32% and ≈35%, respectively, significantly lower than those of the pristine membranes (≈51% for BAF‐QAF and ≈54% for C7‐QAF). The results suggest that PA doping effectively mitigated the thermal degradation of both ammonium and hexyl groups in the membranes.

### 2.3 PA Doping of Quaternized Membranes

The pristine BAF‐QAF and C7‐QAF membranes (both in OH^−^ form) form transparent and flexible membranes after PA doping (Figure S4c,d, Supporting Information). The PA uptake, ADL, and dimensional change of the PA‐doped membranes are presented in **Table** **1** (the data for our previously reported QPAF‐4‐150%PA are also included for comparison).^[^ ^11^ ^]^ The four listed membranes were doped with PA under identical conditions, each exhibiting different PA doping results. The PBI membrane shows the highest PA uptake (242%), presumably because of the higher concentration of PA‐absorbable imidazole groups (5.0 mmol g^−1^) than those of other polymer membranes. Although BAF‐QAF, C7‐QAF, and QPAF‐4 membranes contain the same ammonium concentration (3.0 mmol g^−1^), PA uptake is different, which is in the order of C7‐QAF (180%) >BAF‐QAF (166%) >QPAF‐4 (153%). The concentration of fluorine atoms in the membranes is in the order of C7‐QAF (0 mmol g^−1^) <BAF‐QAF (6.5 mmol g^−1^) <QPAF‐4 (8.7 mmol g^−1^). The low concentration of hydrophobic fluorine atoms might have promoted compatibility with PA. The ADL is in the order of C7‐QAF (9.2) >BAF‐QAF (7.4) >QPAF‐4 (7.2) >PBI (5.3). Despite exhibiting the highest PA uptake, the PBI membrane shows the lowest ADL, probably because of its weak acid–base interactions with PA.

**Table 1:** PA uptake values, ADLs, and swelling ratios of C7‐QAF, QPAF‐4, BAF‐QAF, and PBI membranes.

Membrane	PA uptake [%]	ADL	Swelling ratio [%]		Reference
			Area	Volume	
BAF‐QAF	166	7.4	57	129	This study
C7‐QAF	180	9.2	30	66	This study
QPAF‐4	153	7.2	59	88	[11]
PBI	242	5.3	44	192	This study

**Figure 3:**
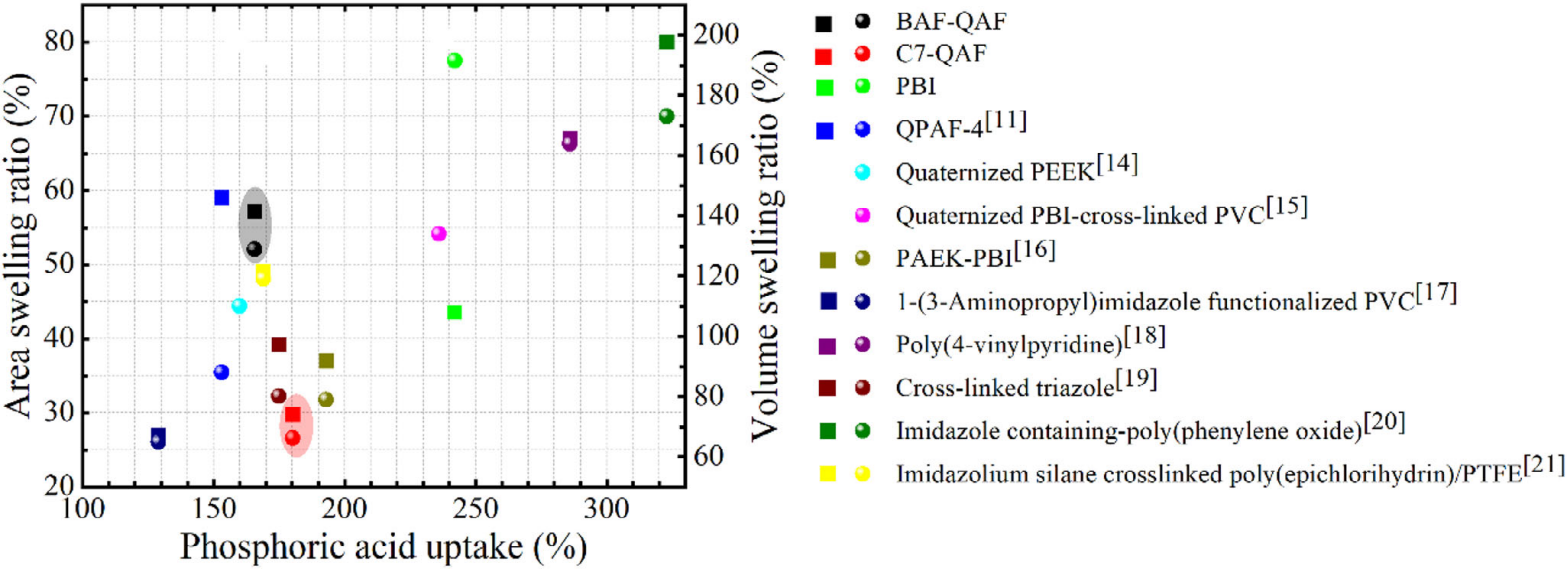
PA uptake, and areal and volumetric swelling ratios of the membranes upon PA‐doping [squares represent areal swelling ratios; balls represent volumetric swelling ratios; PEEK: poly(ether ether ketone); PAEK: poly(aryl ether ketone); PVC: poly(vinyl chloride); PTFE: polytetrafluoroethylene].

The change in the areal dimensions of the membranes via PA‐doping was in the order of C7‐QAF (30%) <PBI (44%) <BAF‐QAF (57%) <QPAF‐4 (59%). The change in the volumetric dimensions follows the order of C7‐QAF (66%) <QPAF‐4 (88%) <BAF‐QAF (129%) <PBI (192%). Among the tested membranes, C7‐QAF exhibits the smallest areal (30%) and volumetric (66%) changes, and these values are nearly half of those of the BAF‐QAF [57% (area) and 129% (volume)], underscoring the high dimensional stability imparted by the fluorine‐free backbone despite high PA uptake. BAF‐QAF membrane shows an areal change (57%) similar to the QPAF‐4 membrane (59%), but its volumetric change (129%) is 41% higher than that of QPAF‐4 (88%) owing to its greater through‐plane swelling, which is associated with high PA uptake. The PBI membrane exhibits similar behavior, showing a low areal change (44%) but the highest volumetric change (192%) among all tested membranes, implying its maximum PA uptake (242%). For further comparison, the PA uptake, ADL, and dimensional changes of several other membranes upon PA‐doping are listed in Table S1 (Supporting Information) and plotted in **Figure** **3**. Fluorine‐free C7‐QAF membrane comparatively outperforms most of the reported PA‐doped membranes (such as membranes containing QA, azolinium, or pyridinium groups, and PBI‐based membranes) with high PA uptake (180%) but low areal (30%) and volumetric (<70%) dimensional changes.^[^ ^11^, ^14^, ^15^, ^16^, ^17^, ^18^, ^19^, ^20^, ^21^ ^]^ doping [squares represent areal swelling ratios; balls represent volumetric swelling ratios; PEEK: poly(ether ether ketone); PAEK: poly(aryl ether ketone); PVC: poly(vinyl chloride); PTFE: polytetrafluoroethylene].

**Figure 4:**
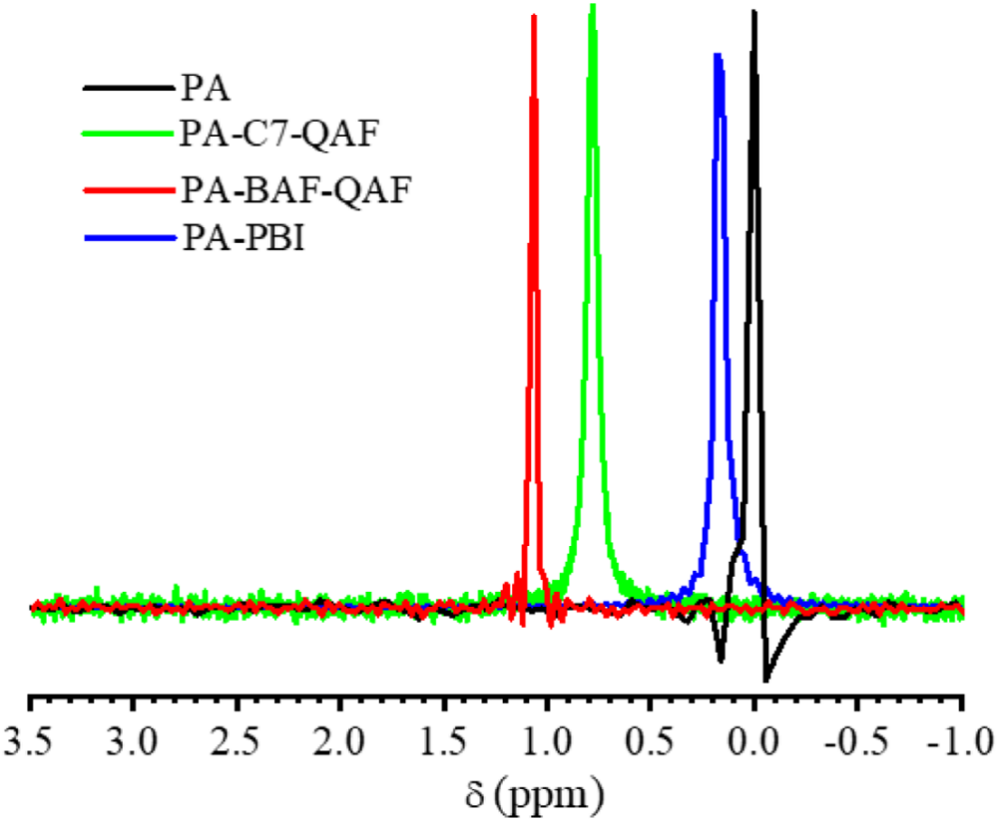
^31^P NMR spectra of pure PA, PA‐C7‐QAF, PA‐BAF‐QAF, and PA‐PBI membranes in DMSO‐*d_6_* at room temperature.

The PA‐doped membranes (PA‐BAF‐QAF, PA‐C7‐QAF, and PA‐PBI) were analyzed via ^31^phosphorus (^31^P) NMR spectroscopy, as shown in **Figure** **4** (the spectrum of pure PA is included for comparison). Pure PA displays a single signal at 0 ppm, whereas the PA‐doped membranes exhibit downfield‐shifted signals at 0.19 ppm (PA‐PBI), 0.78 ppm (PA‐C7‐QAF), and 1.08 ppm (PA‐BAF‐QAF), respectively, owing to the small electron density on the P atoms, resulting from electrostatic interactions with QA groups (BAF‐QAF and C7‐QAF) or imidazolium groups (PBI). The chemical shift exhibited by PA‐BAF‐QAF and PA‐C7‐QAF membranes is >4‐fold larger than that by the PA‐PBI membrane, indicating stronger ion‐pair interactions between QA groups and PA than that between imidazolium groups and PA. This chemical shift is plotted as a function of the ADL (Figure S5, Supporting Information). PA‐C7‐QAF membrane shows a 0.3‐ppm smaller chemical shift than PA‐BAF‐QAF membrane, while its ADL (9.2) is 1.8 higher than that of the latter (7.4). Owing to the higher ADL or PA content of PA‐C7‐QAF membrane, its ion‐pair interaction energy is reduced owing to the distribution of the negative charge of biphosphate ions among additional PA molecules, causing an upfield shift of the ^31^P NMR signal.^[^ ^5^ ^]^ The PA‐PBI membrane with an ADL of 5.3 exhibits a minimum chemical shift, which is similar to that of pure PA, indicating that the PA content of the PA‐PBI membrane does not significantly affect the interaction energy between PA clusters and imidazolium groups.

### 2.4 Mechanical Properties of Membranes

**Figure 5:**
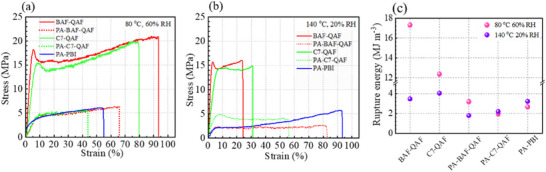
Stress–strain curves of membranes at a) 80 °C and 60% RH, and b) 140 °C and 20% RH. c) Rupture energy of membranes at (80 °C and 60% RH) and (140 °C and 20% RH).

The mechanical properties of pristine BAF‐QAF and C7‐QAF membranes, and PA‐doped PA‐BAF‐QAF, PA‐C7‐QAF, and PA‐PBI membranes were evaluated at (80 °C and 60% RH) and (140 °C and 20% RH). The results are presented in **Figure** **5** and Table S2 (Supporting Information). BAF‐QAF and C7‐QAF membranes exhibit Young's modulus of 3.93 and 2.56 GPa, yield stress of 18.2 and 15.2 MPa, and the maximum strain of 93% and 79%, respectively, at 80 °C and 60% RH (Figure 5a). Although the two exhibit similar molecular weights and identical hydrophilic components, BAF‐QAF is more robust and flexible than C7‐QAF, indicating that the fluorine‐containing BAF moiety strengthens the BAF‐QAF membrane. Moreover, the as‐prepared BAF‐QAF membrane herein is more flexible than our earlier reported BAF‐QAF‐1, ‐2, and ‐3 membranes (maximum strains <15%) under identical testing conditions, presumably owing to its higher IEC (3.00 mmol g^−1^) than those of the reported membranes (≤ 2.82 mmol g^−1^).^[^ ^22^ ^]^ After the PA doping, both PA‐BAF‐QAF and PA‐C7‐QAF membranes show inferior mechanical properties with Young's modulus decreasing to 0.57 and 0.61 GPa, yield stress decreasing to 4.4 and 4.6 MPa, and maximum strain decreasing to 66% and 43%, respectively. These changes underscore the plasticizing effect of PA on the membranes. Even after mechanical attenuation via PA doping, the PA‐BAF‐QAF membrane is more flexible, with its maximum strain 23% larger than that of the PA‐C7‐QAF membrane. It outperforms PA‐PBI membrane, which had a Young's modulus of 0.35 GPa, yield stress of 3.8 MPa, and maximum strain of 55%. The higher rupture energy (Figure 5c) of the PA‐BAF‐QAF membrane (3.18 MJ m⁻^3^) compared with those of PA‐C7‐QAF (1.96 MJ m⁻^3^) and PA‐PBI (2.66 MJ m⁻^3^) at 80 °C and 60% RH also highlights its superior mechanical strength. Notably, PA‐C7‐QAF and PA‐PBI membranes might have lost some PA during measurements (discussed in detail as the weight loss observed during RH cycling), which can lightly mitigate the plasticizing effect.

At high temperatures (140 °C) and low humidity (20% RH; Figure 5b), BAF‐QAF and C7‐QAF membranes exhibit inferior mechanical properties with Young's modulus decreasing from 3.93 and 2.56 GPa to 3.84 and 2.45 GPa, yield stress from 18.2 and 15.2 MPa to 15.6 and 14.3 MPa, and their maximum strain from 93% and 79% to 24% and 31%, respectively. Therefore, the corresponding rupture energies of BAF‐QAF and C7‐QAF decrease from 17.31 and 12.36 MJ m⁻^3^ (80 °C; 60% RH) to 3.47 and 4.04 MJ m⁻^3^ (140 °C; 20% RH), respectively. BAF‐QAF membrane exhibits a lower maximum strain and rupture energy than C7‐QAF at 140 °C and 20% RH, indicating that the hydrocarbon backbone of C7‐QAF is less sensitive to temperature and humidity than the fluorine‐containing backbone of BAF‐QAF. PA‐BAF‐QAF, PA‐C7‐QAF, and PA‐PBI membranes show improved mechanical properties with their maximum strain increasing from 66%, 43%, and 55% (80 °C; 60% RH) to 83%, 56%, and 93% (140 °C; 20% RH), respectively. Consequently, the rupture energy of PA‐BAF‐QAF membrane decreases from 3.18 MJ m⁻^3^ (80 °C; 60% RH) to 1.79 MJ m⁻^3^ (140 °C; 20% RH), while those of PA‐C7‐QAF and PA‐PBI membranes increased from 1.96 and 2.66 MJ m⁻^3^ (at 80 °C and 60% RH) to 2.20 and 3.11 MJ m⁻^3^ (at 140 °C 20% RH), respectively. These results imply that the fluorine‐free polymer backbones in PA‐doped membranes are highly mechanically stable to increasing temperature and decreasing humidity.

### 2.5 Morphology

**Figure 6:**
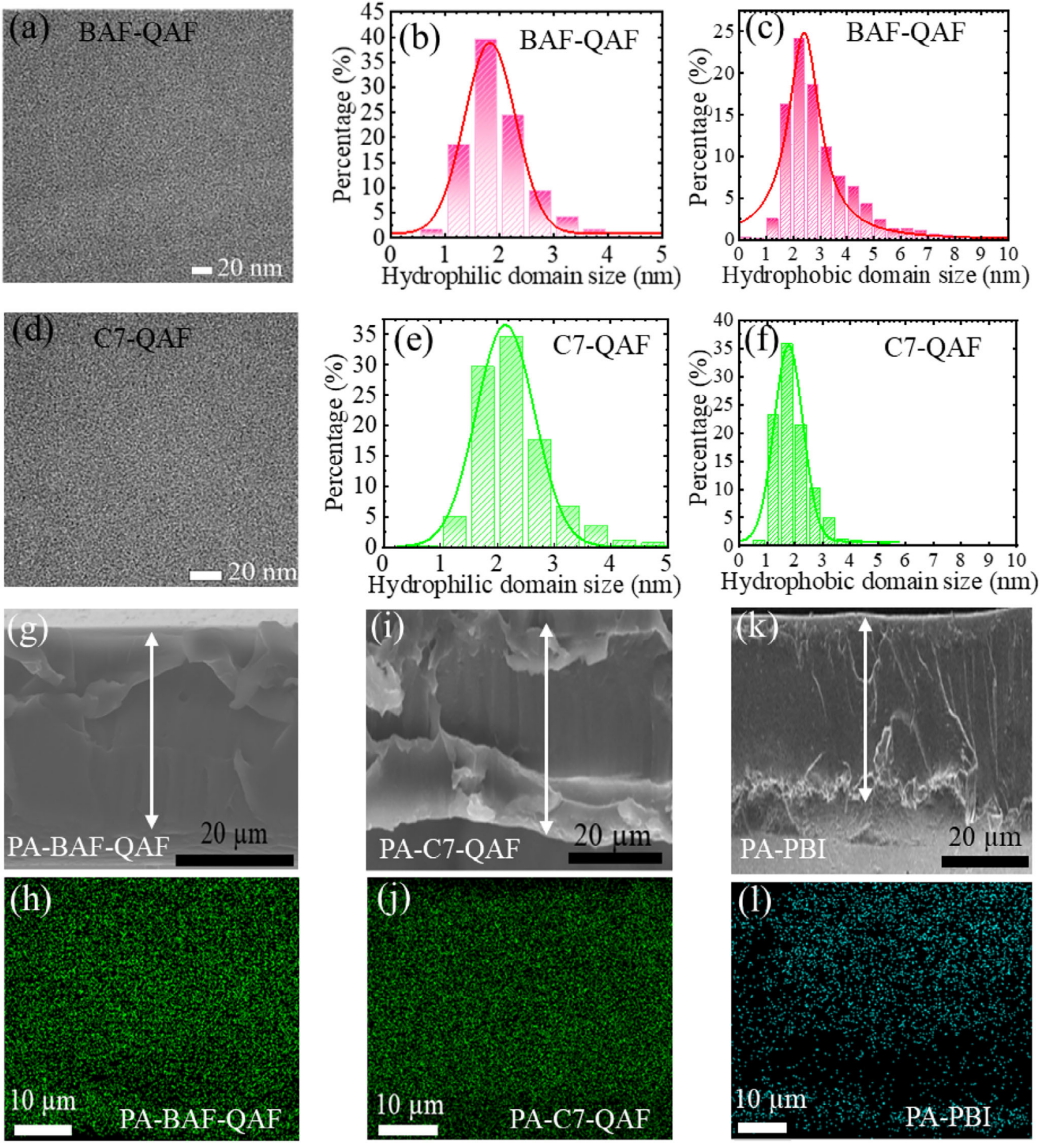
TEM images of a) BAF‐QAF and d) C7‐QAF membranes. b,c,e,f) Hydrophilic and hydrophobic domain size distributions. Cross‐sectional SEM images of g) PA‐BAF‐QAF, i) PA‐C7‐QAF, and k) PA‐PBI (white arrows indicate the cross‐sections of the membranes. h,j,l) Phosphorus elemental mappings.

The phase‐separated morphologies of pristine BAF‐QAF and C7‐QAF membranes stained with platinum chloride anion (PtCl_4_^2−^) were analyzed via transmission electron microscopy (TEM), as shown in **Figure** **6**a,d. The dark regions represent hydrophilic clusters containing QA groups and the bright areas represent hydrophobic domains. The sizes of hydrophilic and hydrophobic domains were measured from >800 spots, and their size distributions are presented in Figure 6b,c,e,f, respectively. The hydrophilic domain size of the BAF‐QAF membrane is 1.83 ± 0.06 nm, which is 0.31 nm smaller than that of the C7‐QAF membrane (2.14 ± 0.14 nm). As their IEC values are the same, C7 groups as the hydrophobic components probably contributed less to the highly developed hydrophilic clusters. Large hydrophilic clusters induce high PA doping degrees. In contrast, the hydrophobic domain size of BAF‐QAF (2.41 ± 0.14 nm) is 0.63 nm larger than that of C7‐QAF (1.78 ± 0.07 nm).

The cross‐sectional images of the PA‐BAF‐QAF, PA‐C7‐QAF, and PA‐PBI membranes were analyzed via scanning electron microscopy (SEM) and energy‐dispersive X‐ray spectroscopy (Figure 6g–l). The scatter‐distributed morphologies of all membranes are confirmed without the bulk localization of P atoms throughout the cross‐sectional area (Figure 6h,j,l), indicating that PA is successfully and homogeneously absorbed in the interior of membranes. Under vacuum conditions during SEM measurements, absorbed PA might have evaporated to a certain extent. However, PA‐BAF‐QAF and PA‐C7‐QAF membranes exhibit higher P concentrations than the PA‐PBI membrane, which is similar to the above‐mentioned conclusion about the higher affinity of aliphatic ammonium groups for PA.

**Figure 7:**
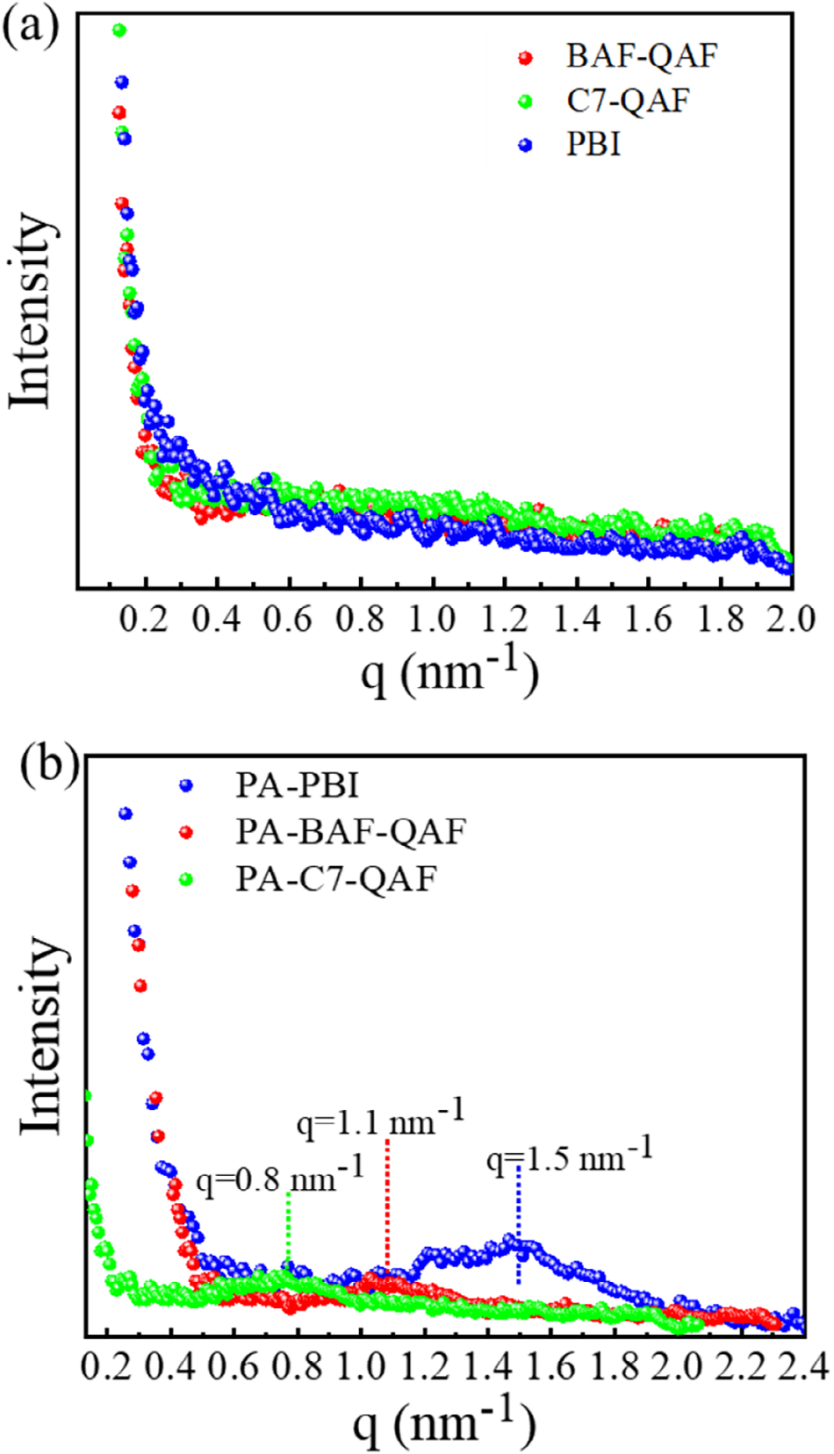
SAXS patterns of a) BAF‐QAF, C7‐QAF, and PBI membranes and b) PA‐BAF‐QAF, PA‐C7‐QAF, and PA‐PBI membranes.

The periodic structure of the membranes based on phase separation was analyzed via small‐angle X‐ray scattering (SAXS; **Figure** **7**). No peaks are observed in the SAXS curves of BAF‐QAF, C7‐QAF, and PBI membranes, indicating that these membranes do not contain a periodic structure at the nanometer scale. In contrast, PA‐BAF‐QAF, PA‐C7‐QAF, and PA‐PB membranes exhibit broad and minor peaks at the q values of 1.1, 0.8, and 1.5 nm^−1^, which correspond with the d‐spacings of 5.7, 7.9, and 4.2 nm, respectively. Assuming the d‐spacing is the average size of PA ionic clusters, PA‐BAF‐QAF and PA‐C7‐QAF exhibit ionic clusters with sizes ≥1.5 nm larger than those in the PA‐PBI membrane. The large ionic cluster sizes of PA‐BAF‐QAF and PA‐C7‐QAF membranes might be related to their high PA doping levels (ADLs of PA‐BAF‐QAF, PA‐C7‐QAF, and PA‐PBI are 7.4, 9.2, and 5.3, respectively).

### 2.6 Weight Change and Proton Conductivity during Humidity Cycling Tests

**Figure 8:**
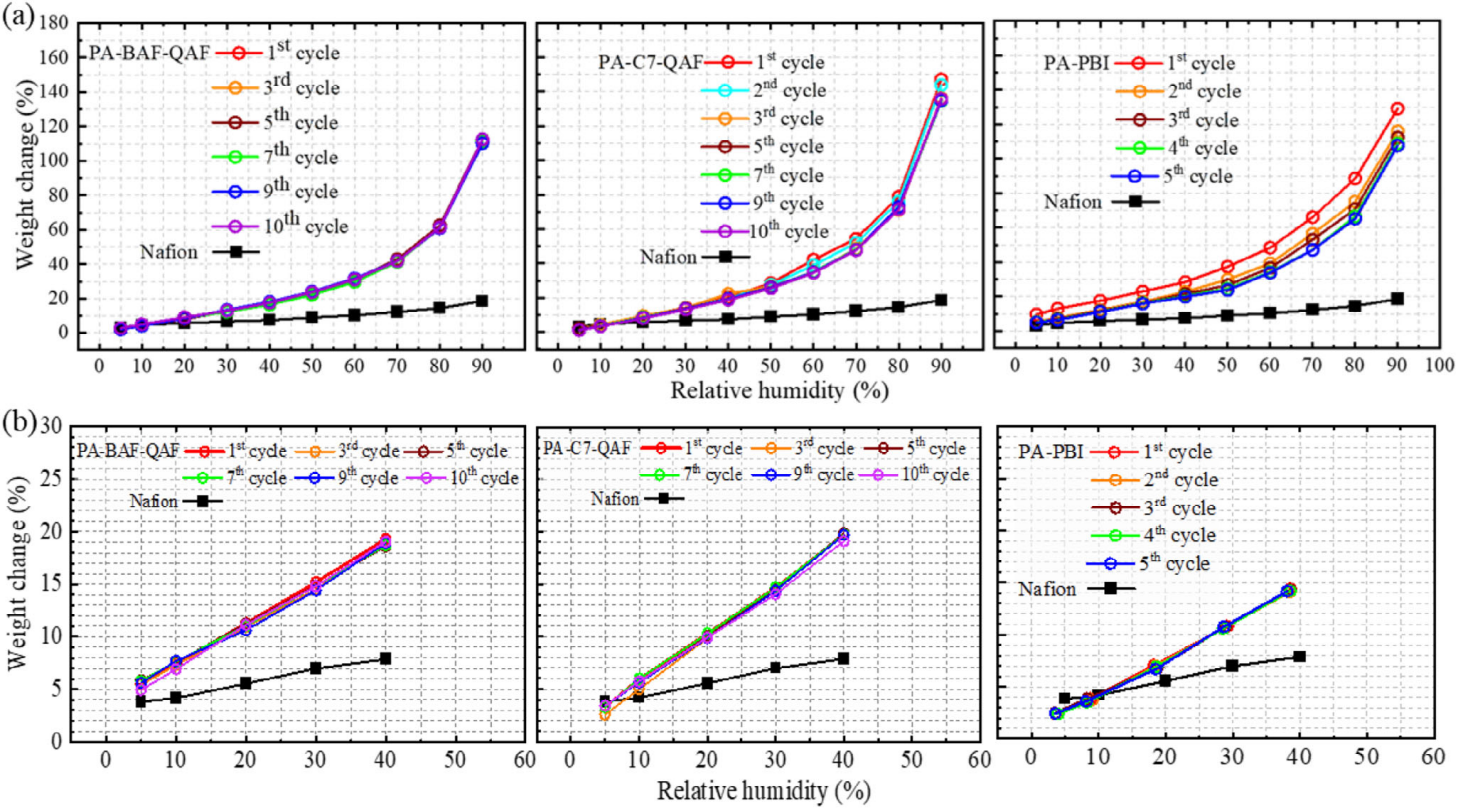
Weight change of PA‐doped membranes during RH cycling at a) 80 and b) 120 °C.

PA‐BAF‐QAF, PA‐C7‐QAF, and PA‐PBI membranes were subjected to humidity cycling at 80 (**Figure** **8**a) and 120 °C (Figure 8b) to monitor the changes in their weight, and the data of the Nafion membrane (Nafion NR‐211) were used for comparison. The weights of all membranes increase as humidity increases from 5% to 90% RH owing to water uptake. The increase in the weight of the membranes was larger than that of the Nafion membrane at any RH. The weight change did not change but was repeatable in the humidity cycling of the PA‐BAF‐QAF membrane, indicating no practical loss of PA. PA‐C7‐QAF membrane shows more weight increase (≥ 136%; 90% RH) than PA‐BAF‐QAF (110%; 90% RH) at high humidity, probably because of its high PA doping level (PA uptake = 180%) than PA‐BAF‐QAF (PA uptake = 166%). The PA‐C7‐QAF membrane exhibited a slight decrease in weight change at high humidities (≥ 60% RH) as repeating the humidity cycles; the weight change at 90% RH was 147% in the first cycle and 135% in the third cycle. This result implies the leaching of a certain amount of PA from, probably because the high ADL (9.2) weakens ion‐pair interactions. The increase in the weight of the PA‐PBI membrane is smaller than those of the other two membranes during humidity cycling, particularly during the first and second cycles, and the weight change decreases at all tested humidity levels. At high humidity (90% RH), the weight change of the membrane is 129% in the first cycle and decreases to 116% in the second cycle, 112% in the third cycle, 109% in the fourth cycle, and 108% in the fifth cycle. This large decrease in the weight change of PA‐PBI membrane is attributed to its weak acid–base interactions, which are more pronounced than ion‐pair interactions. At 120 °C, the PA‐BAF‐QAF, PA‐C7‐QAF, and PA‐PBI membranes exhibit relatively stable weight changes during humidity cycles from 5% to 40% RH, indicating strong PA retention at high temperatures and low humidity levels.

**Figure 9:**
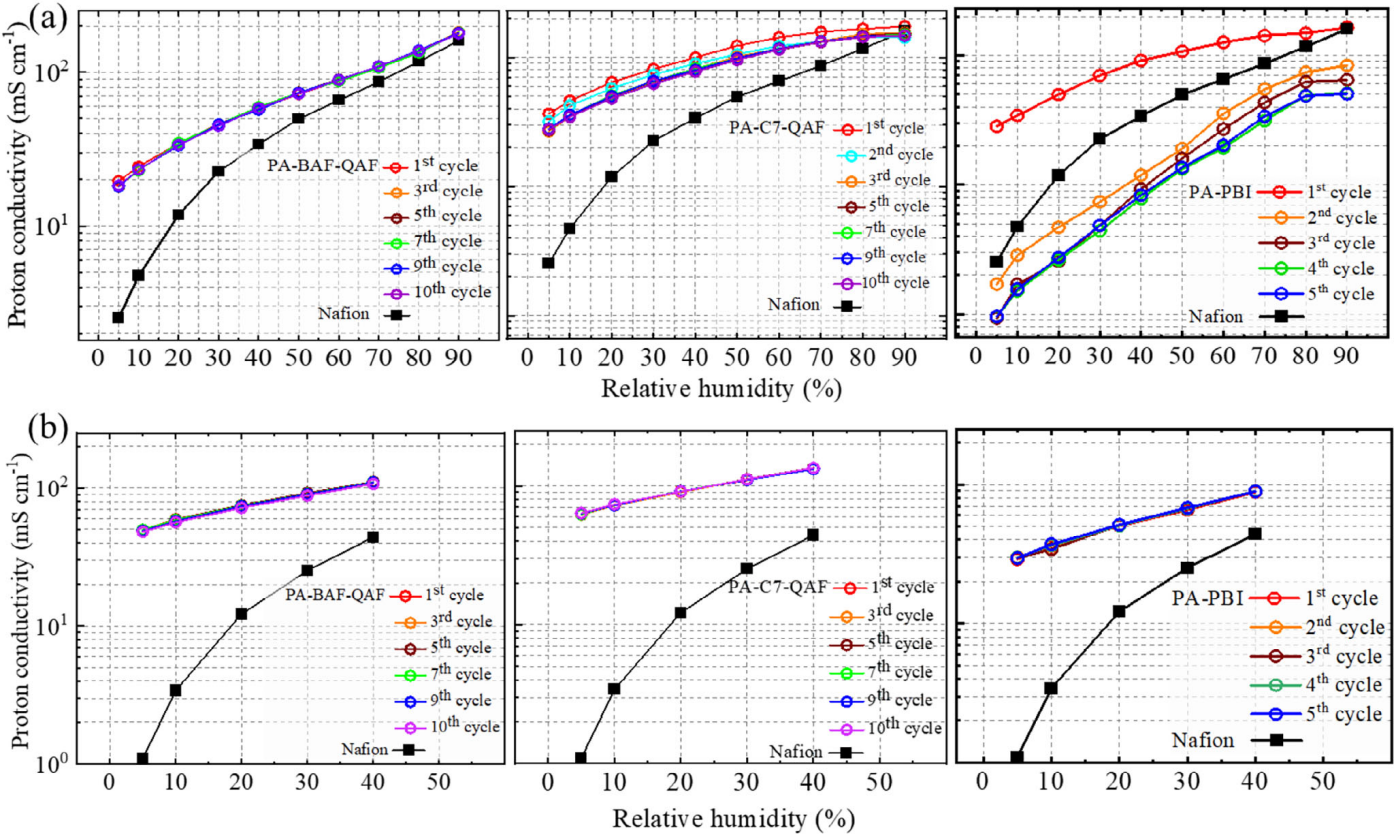
Proton conductivity of PA‐doped membranes during RH cycling at a) 80 and b) 120 °C.

The proton conductivity of PA‐doped membranes was measured during humidity cycling at 80 (**Figure** **9**a) and 120 °C (Figure 9b). At 80 °C, PA‐BAF‐QAF and PA‐C7‐QAF membranes exhibit high proton conductivity (≥ 18 mS cm^−1^ at 5% RH) with less dependency on humidity compared with the Nafion membrane (2.5 mS cm^−1^ at 5% RH). PA‐BAF‐QAF exhibits stable proton conductivity from 18.0 mS cm^−1^ (5% RH) to 178.4 mS cm^−1^ (90% RH) without an obvious decrease during all humidity cycles owing to its high PA retainability. The conductivity of PA‐C7‐QAF slightly decreases from 36.6 mS cm^−1^ (5% RH) and 174.7 mS cm^−1^ (90% RH) in the first cycle to 27.7 mS cm^−1^ (5% RH) and 148.6 mS cm^−1^ (90% RH) in the third cycle, after which the conductivity does not decrease. Minor PA loss during the first and second cycles results in a reduction in initial conductivity. PA‐BAF‐QAF membrane exhibits a proton conductivity of 178.4 mS cm^−1^ at 90% RH, which is higher than that of the PA‐C7‐QAF membrane (148.6 mS cm^−1^). At low humidity (5% RH), the PA‐BAF‐QAF membrane exhibits lower proton conductivity (18.0 mS cm^−1^) than the PA‐C7‐QAF membrane (27.7 mS cm^−1^), indicating that the PA‐C7‐QAF membrane is more proton conductive than the PA‐BAF‐QAF membrane at low humidity, attributable to its larger PA‐containing ionic clusters (7.9 vs 5.7 nm of PA‐BAF‐QAF), forming more developed proton‐transporting channels. PA‐PBI membrane exhibits proton conductivity values of 28.3 and 165.0 mS cm^−1^ at 5% and 90% RH in the first cycle, respectively, which are comparable to those of PA‐BAF‐QAF and PA‐C7‐QAF. However, the conductivity of PA‐PBI membranes decreases significantly to 1.7 and 83.9 mS cm^−1^ at 5% and 90% RH, respectively, in the second cycle, which is lower than that of the Nafion membrane. By the fourth cycle, the conductivity is stabilized at 1.0 and 50.5 mS cm^−1^ at 5% and 90% RH, respectively, losing >68% of its initial conductivity. This significant decline in conductivity is attributed to severe PA loss at high humidity levels. At 120 °C, the three PA‐doped membranes do not lose their proton conductivities during humidity cycling, as also supported by the absence of PA loss at high temperatures and low humidity levels. The proton conductivity follows the mentioned order: PA‐C7‐QAF (63.6 mS cm^−1^ at 5% RH and 134.4 mS cm^−1^ at 40% RH) > PA‐BAF‐QAF (48.9 mS cm^−1^ at 5% RH and 110.0 mS cm^−1^ at 40% RH) >PA‐PBI (29.7 mS cm^−1^ at 5% RH and 89.3 m^−1^ at 40% RH). This order agrees with the size of the PA ionic clusters [PA‐C7‐QAF (7.9 nm) >PA‐BAF‐QAF (5.7 nm) >PA‐PBI (4.2 nm)], where large PA ionic clusters contribute to high proton conductivity.

### 2.7 Temperature‐Dependent Proton Conductivity

**Figure 10:**
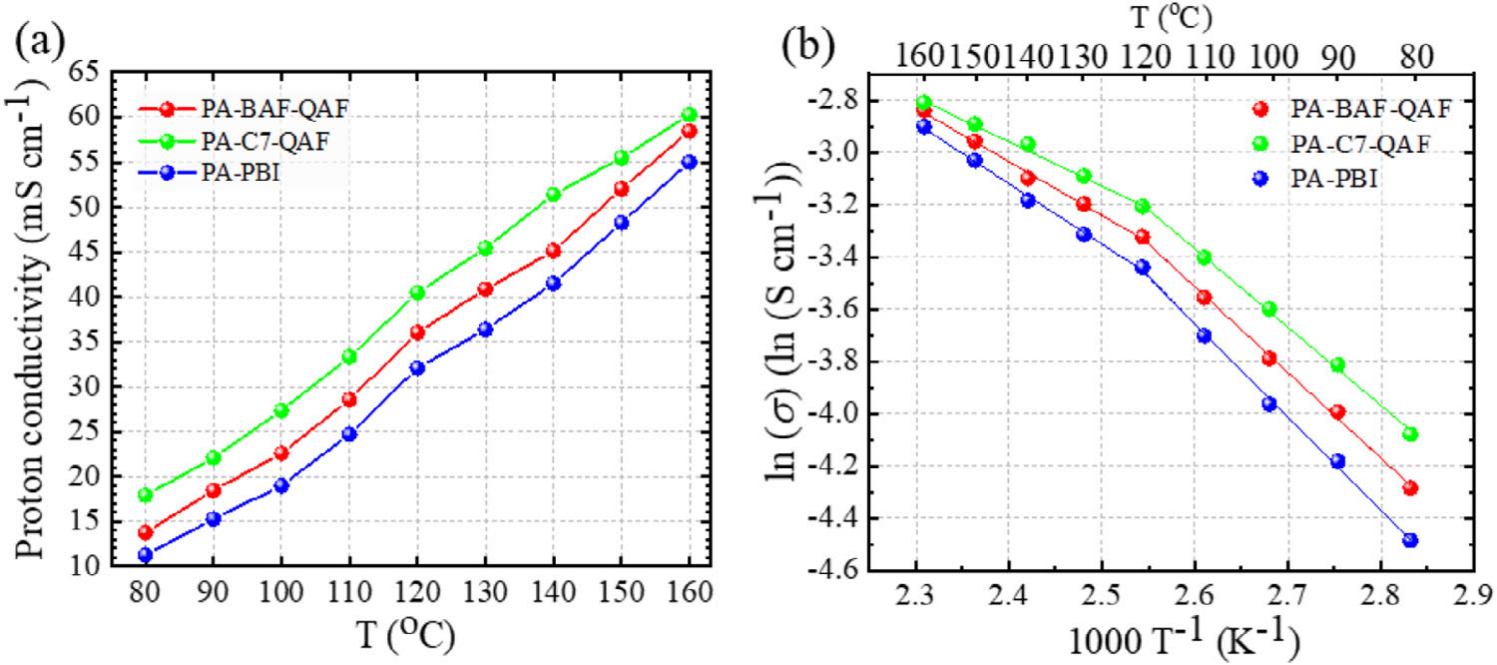
a) Temperature‐dependent proton conductivity and b) Arrhenius plots of the proton conductivity of PA‐doped membranes.

The proton conductivity of PA‐doped membranes was measured under anhydrous conditions in the temperature range of 80–160 °C, as illustrated in **Figure** **10**a. The proton conductivity follows the mentioned order: PA‐C7‐QAF (17.9 mS cm^−1^ at 80 °C and 60.3 mS cm^−1^ at 160 °C) >PA‐BAF‐QAF (13.8 mS cm^−1^ at 80 °C and 58.4 mS cm^−1^ at 160 °C) >PA‐PBI (11.3 mS cm^−1^ at 80 °C and 55.0 mS cm^−1^ at 160 °C). This order is similar to the order observed under humidified conditions during humidity cycling (5% to 40% RH) at 120 °C and corresponds to the size of PA‐containing ionic clusters. Conductivity exhibits an Arrhenius‐type dependence on temperature, where two different slopes are observed at a transition point of ≈120 °C for all three PA‐doped membranes (Figure 10b). The apparent activation energies (E_a_; Table S3, Supporting Information) are 25–30 and 14–19 kJ mol^−1^ at low and high temperatures, respectively. This change is likely attributed to water evaporation from the membranes, which alters the proton transport mechanism, shifting it from the vehicle mechanism to the Grotthuss mechanism at elevated temperatures.^[^ ^23^, ^24^ ^]^ A similar temperature dependence of conductivity is observed for other PA‐doped blend membranes such as PBI/poly(4‐vinylpyridine), PBI/polyimide, and PBI/polyvinylpyrrolidone.^[^ ^25^, ^26^ ^]^ Melchior et al. studied proton conduction in a PA–water system, which revealed that at low temperatures and high water contents, conductivity was primarily governed by the vehicular mechanism. As the temperature increased and water content decreased, phosphate species aggregated via strong hydrogen bonding, enhancing structural proton diffusion via hopping, which promoted proton transport and lowered the *E_a_* for proton conduction.^[^ ^27^ ^]^

### 2.8 DFT Calculations

**Figure 11:**
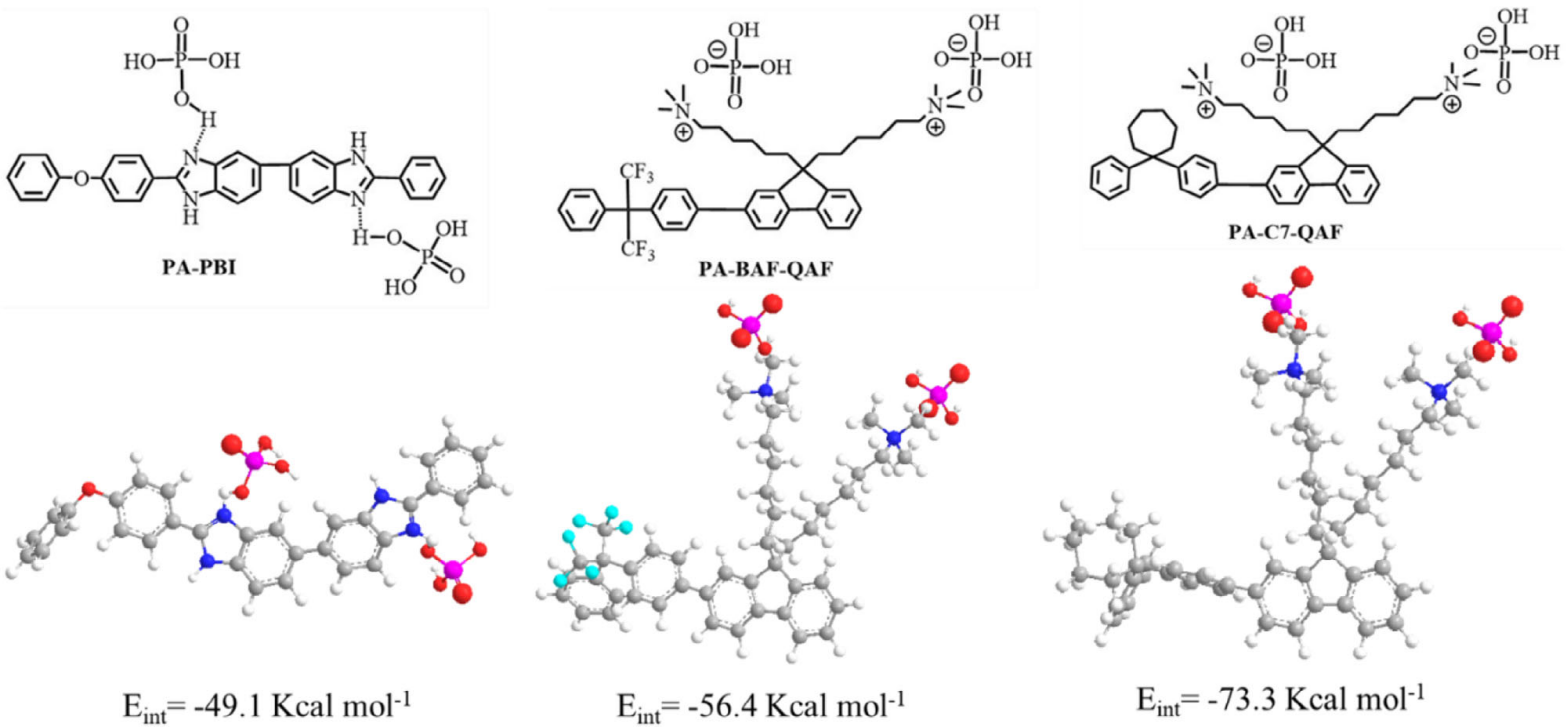
DFT calculations of interaction energies (E_int_) for the repeating units of PA‐PBI, PA‐BAF‐QAF, and PA‐C7‐QAF copolymers with PA. C, gray; H, white; N, blue; O, red; F, cyan; P, pink.

To better understand the differences in the stability of PA‐doped membranes, DFT calculations using DMol^3^ software (BIOVIA Materials Studio, version 2023)^[^ ^28^ ^]^ were performed for the repeating units of PA‐PBI, PA‐BAF‐QAF, and PA‐C7‐QAF copolymers for calculating their interaction energies (E_int_) with PA (**Figure** **11**). The calculation was performed using GGA^[^ ^29^ ^]^ and the PBE functional with a DNP basis set. PA‐C7‐QAF exhibits the strongest E_int_ of −73.3 kcal mol^−1^, followed by PA‐BAF‐QAF (−54.6 kcal mol^−1^) and PA‐PBI (−41.9 kcal mol^−1^). The higher interaction energy of QA‐based copolymers (C7‐QAF and BAF‐QAF) with PA compared to benzimidazolium‐based polymers is presumably attributed to the strong basicity of the ammonium ion (pKa = 9.25)^[^ ^5^ ^]^ than that of the benzimidazolium ion (pKa = 5.5),^[^ ^30^ ^]^ which conforms with the interaction strength estimated from ^31^P NMR spectra (Figure 4). The interaction energy follows the order of PA‐C7‐QAF >PA‐BAF‐QAF >PA‐PBI, agreeing with the order of the measured proton conductivity under low or no humidified conditions (Figures 9 and 10). The high E_int_ of PA‐BAF‐QAF and PA‐C7‐QAF are also responsible for their high PA retention (Figure 8).

### 2.9 Fuel Cell Performance

**Figure 12:**
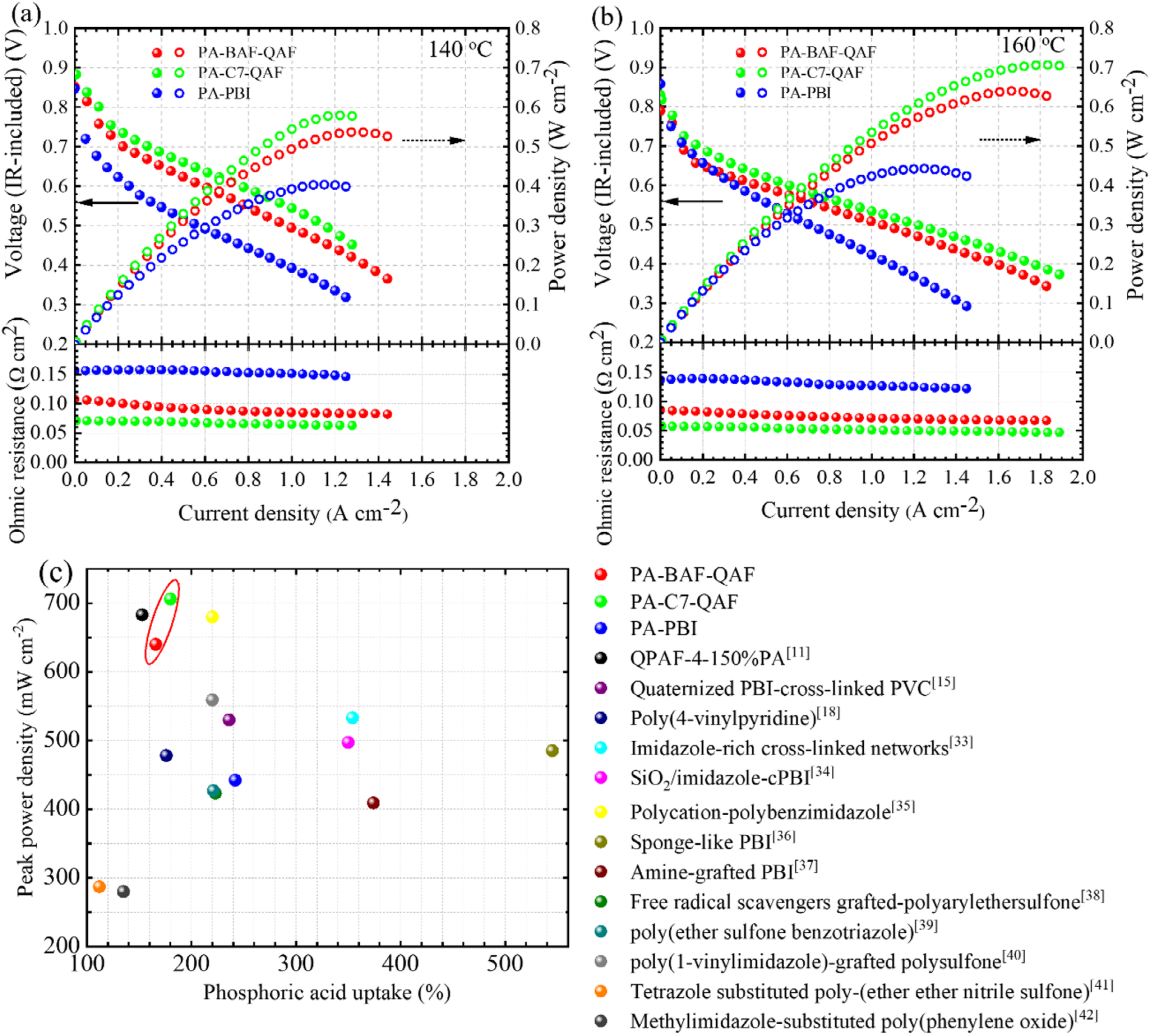
Polarization (IR‐included), power density, and ohmic resistance curves of PA‐doped membranes evaluated at a) 140 and b)160 °C. c) Comparison of the peak power density as a function of the PA uptake of PA‐doped membranes.

The performance of the PA‐doped membranes in fuel cells was evaluated at 140 and 160 °C under ambient pressure and anhydrous conditions. Dry H_2_ (80 mL min^−1^) and O_2_ (160 mL min^−1^) were supplied to the anode and cathode, respectively. The ohmic resistance (IR) with polarization and power density curves are shown in **Figure** **12**a,b. At 140 °C, the open‐circuit voltages (OCVs) of PA‐C7‐QAF (42‐µm thick), PA‐BAF‐QAF (33‐µm thick), and PA‐PBI (56‐µm thick) cells are 0.883, 0.852, and 0.847 V, respectively. The high OCVs of PA‐C7‐QAF and PA‐BAF‐QAF cells, despite their lower thickness than the PA‐PBI cell, imply high gas barrier properties. The peak power densities of cells also follow the same order with values of 0.579, 0.537, and 0.404 W cm^−2^ for PA‐C7‐QAF, PA‐BAF‐QAF, and PA‐PBI cells, respectively. The superior performance of PA‐C7‐QAF and PA‐BAF‐QAF cells than the PA‐PBI cell is probably owing to their enhanced proton conductivities at high temperatures. The ohmic resistance of the cells follows the mentioned order: PA‐C7‐QAF (0.06–0.07 Ω cm^2^) <PA‐BAF‐QAF (0.08–0.11 Ω cm^2^) <PA‐PBI (0.15–0.16 Ω cm^2^), which corresponds to the aforementioned trend for the proton conductivity of the membranes at 120 °C and low humidity levels. At 160 °C, the OCVs of PA‐C7‐QAF, PA‐BAF‐QAF, and PA‐PBI cells are 0.830, 0.790, and 0.858 V, respectively. The slight reduction in the OCVs of PA‐C7‐QAF and PA‐BAF‐QAF cells is attributed to increased gas permeation caused by the softening of the aliphatic ammonium group–containing membranes at elevated temperatures. The ohmic resistance of the cells is slightly lower than that at 140 °C (0.05–0.06 Ω cm^2^, PA‐C7‐QAF; 0.07–0.08 Ω cm^2^, PA‐BAF‐QAF; and 0.12–0.14 Ω cm^2^, PA‐PBI), implying improved proton conductivity. Accordingly, the peak power density is higher at 160 °C with values of 0.706, 0.640, and 0.442 W cm^−2^ for PA‐C7‐QAF, PA‐BAF‐QAF, and PA‐PBI cells, respectively. High power density at high temperatures is a well‐documented behavior, as reported by Lee and others for PA‐doped PEMs, where the enhanced reaction kinetics of the oxygen reduction reaction is responsible.^[^ ^31^, ^32^ ^]^ Compared to the previously reported PA‐doped membranes, PA‐C7‐QAF and PA‐BAF‐QAF membranes exhibit higher peak power densities with low PA uptakes (Figure 12c; Table S4, Supporting Information).^[^ ^11^, ^15^, ^18^, ^33^, ^34^, ^35^, ^36^, ^37^, ^38^, ^39^, ^40^, ^41^, ^42^ ^]^ PA‐C7‐QAF membrane achieves a peak power density of 0.706 W cm^−2^, surpassing PA‐BAF‐QAF membrane by 0.066 W cm^−2^ and our earlier reported QPAF‐4‐150%PA membrane by 0.023 W cm^−2^, whose proton conductivity (52.0 mS cm^−1^ at 160 °C) is lower than that of PA‐C7‐QAF (60.3 mS cm^−1^ at 160 °C). These results highlight PA‐C7‐QAF as a promising candidate for fluorine‐free, polyphenylene‐based PA‐doped membranes in HT‐FCs.

The durability of the PA‐C7‐QAF cell was evaluated at a constant current density of 0.15 A cm^−2^ at 140 °C (Figure S6, Supporting Information), and the data of the PA‐PBI cell were included for comparison. The voltage of the PA‐PBI cell was initially 0.660 V and gradually decreased to 0.629 V after 7.6 h, followed by significant voltage fluctuations until the end of the measurement after 12.3 h, at which the voltage was 0.566 V. The ohmic resistance of the PA‐PBI cell was initially 0.10 Ω cm^2^ and remained nearly constant for 7.6 h. However, it slightly increased to 0.11 Ω cm^2^ and eventually increased to 0.12 Ω cm^2^ at 12.3 h. The ohmic resistance increase and voltage drop of the PA‐PBI cell are attributed to PA leaching from the membrane to the catalyst layer. Similar behavior was reported by Zhang and other groups for PA‐doped PBI membranes in HT‐FCs.^[^ ^36^, ^43^, ^44^ ^]^ In contrast, the voltage of the PA‐C7‐QAF cell was initially 0.735 V, followed by a slight decrease to 0.730 V within the first 0.3 h and a gradual decrease to 0.729 V after 33.3 h, indicating a minor average voltage decay rate of 30 µV h^−1^ after the initial drop. The ohmic resistance of the PA‐C7‐QAF cell was initially 0.08 Ω·cm^2^ and slightly decreased to 0.07 Ω cm^2^ after 33.3 h, indicating the high PA retention of the membrane. The durability based on the cell voltage outperforms those of the previously reported PA‐doped membranes, including QPAF‐4‐150%PA (200 µV h^−1^) and other nitrogen heterocycle–containing membranes such as Im‐Pa‐PPO‐20%/130%PA (50 µV h^−1^), 235%PA@DPBI‐10PVBC (780 µV h^−1^), and PA‐doped PPy (270 µV h^−1^) under identical test conditions.^[^ ^11^, ^45^, ^46^, ^47^ ^]^ The exceptional performance reflects the strong voltage retention of the PA‐C7‐QAF cell, benefiting from its robust PA retainability.

## 3 Conclusion

Herein, two polyphenylene‐based quaternized membranes (BAF‐QAF and C7‐QAF) featuring distinct hydrophobic moieties (BAF and C7) and fluorenyl groups with pendant QA groups were successfully prepared and doped with PA to investigate the effect of the hydrophobic components on the physical/chemical properties of the resulting PEMs and HT‐FC performance. The TGA analyses revealed the thermal stability of BAF‐QAF and C7‐QAF without weight loss up to 180 °C, confirming their applicability in HT‐FCs. Compared with the QPAF‐4‐150%PA membrane prepared in our earlier study, PA‐BAF‐QAF (166%) and PA‐C7‐QAF (180%) membrane exhibited higher PA uptakes under identical conditions. The lower fluorine contents of C7‐QAF (0 mmol g^−1^) and BAF‐QAF (6.5 mmol g^−1^) compared with that of the QPAF‐4 (8.7 mmol g^−1^) membrane might have promoted their compatibility with PA. The significantly higher ADL values of C7‐QAF (9.2) and BAF‐QAF (7.4) than that of PBI (5.3), implied their stronger ion‐pair interactions with PA, while PBI primarily relied on acid–base interactions. PA‐BAF‐QAF‐ and PA‐C7‐QAF demonstrated excellent stretchability, achieving maximum strains of 66% and 43% at 80 °C and 60% RH, and 83% and 56% at 140 °C and 20% RH, respectively. During humidity cycle testing at 80 °C, PA‐BAF‐QAF and PA‐C7‐QAF membranes exhibited robust PA retention, maintaining 100% and 85% of their initial conductivity at 90% RH after 10 cycles, respectively, outperforming the PA‐PBI membrane which exhibited <31% of its initial conductivity after 3 cycles. Benefiting from the well‐developed microphase separation, large PA ionic clusters (7.9 nm for PA‐C7‐QAF and 5.7 nm for PA‐BAF‐QAF), and strong E_int_ with PA, the PA‐C7‐QAF and PA‐BAF‐QAF membrane exhibited superior proton conductivities of 134.4 and 110.0 mS cm^−1^ at 120 °C (40% RH) and 60.3 and 58.4 mS cm^−1^ at 160 °C, respectively. The excellent PA retention and high proton conductivity of PA‐C7‐QAF and PA‐BAF‐QAF membranes enabled their high performance in HT‐FCs with peak power densities of 0.579 and 0.537 W cm^−2^ at 140 °C and 0.706 and 0.640 W cm^−2^ at 160 °C, respectively. The ohmic resistances associated with the proton conductivity of the membranes were low (≈0.07 and ≈0.10 Ω cm^2^) at 140 °C and (≈0.06 and ≈0.08 Ω cm^2^) 160 °C, respectively. The PA‐C7‐QAF membrane survived durability testing at a constant current density of 0.15 A cm^−2^ at 140 °C for 33.3 h, with an average voltage decay of 30 µV h^−1^ after an initial drop. This study reveals the importance of hydrophobic components in aromatic polymer‐based, ion‐pair interacting membranes as PEMs for applications in HT‐FCs.

## 4 Experimental Section

Materials, synthesis of PBI polymer (Scheme S1 and Figure S1, Supporting Information), and measurements details are provided in the Supporting Information.

### Synthesis of C7‐AF and C7‐QAF Polymers

Before polymer synthesis, the C7 monomer was synthesized first via acid‐catalyzed condensation and Sandmeyer reactions (Scheme S2, Supporting Information). Figure S2 (Supporting Information) shows the proposed structures. To synthesize the C7‐AF polymer, a 100‐mL three‐necked flask equipped with a condenser, nitrogen inlet/outlet, and magnetic stirring bar was charged with C7 (0.42 g, 1.32 mmol), 6,6′‐(2,7‐dichloro‐9H‐fluorene‐9,9‐diyl)bis(N,N‐dimethylhexan‐1‐amine) (AF; 0.73 g, 1.50 mmol), 2.2ʹ‐bipyridine (BPY; 1.39 g, 8.87 mmol), and *N,N*‐dimethylacetamide (DMAc; 8 mL). The mixture was heated to 80 °C, followed by the addition of bis(1,5‐cyclooctadiene)nickel(0) (Ni(cod)_2_; 2.32 g, 8.45 mmol). The polymerization reaction was performed at 80 °C for 3 h. The reaction mixture was then washed once with concentrated hydrochloric acid (conc. HCl):methanol = 1:1, v/v for 8 h, followed by washing thrice with pure water (2 h each time). After drying in a vacuum oven at 60 °C overnight, the yellow C7‐AF polymer was obtained (yield: 0.98 g, 98%).

Subsequently, the quaternization of the C7‐AF polymer was conducted to prepare the C7‐QAF polymer. C7‐AF (0.98 g) was dissolved in DMAc (20 mL) in a 100‐mL flask. After a homogeneous solution was obtained, dimethyl sulfate (15 mL) was added dropwise. The reaction was conducted at 50 °C for 48 h and quenched by slowly adding the mixture to excess water. The obtained polymer was filtered, washed thrice with pure water, and dried in a vacuum at 60 °C overnight (yield: 1.30 g, 97%). The ion exchange capacity (IEC) of the C7‐QAF polymer was 3.00 mmol g^−1^.

### Synthesis of BAF‐AF and BAF‐QAF Polymers

The synthesis of BAF‐AF and its quaternized polymer BAF‐QAF (IEC = 3.00 mmol g^−1^) was similar to that of C7‐AF and C7‐QAF polymers, except that the C7 monomer was replaced with the BAF monomer. Details on the synthesis and structural verification of BAF‐AF and BAF‐QAF are provided in the earlier study.^[^ ^22^ ^]^

### Density Functional Theory (DFT) Calculations

DFT calculations were performed for the repeating units of PA‐PBI, PA‐BAF‐QAF, and PA‐C7‐QAF copolymers using DMol^3^ software (BIOVIA Materials Studio, version 2023).^[^ ^28^ ^]^ The geometric optimizations of the PA–base complex were performed using the generalized gradient approximation (GGA)^[^ ^29^ ^]^ and the Perdew‐Burke‐Ernzerhof (PBE) functional with the double numerical basis set with polarization (DNP) with fine‐quality settings (convergence criteria = 2 × 10^−5^ Ha, maximum force = 0.004 Ha Å^−1^, maximum iteration = 500 cycles, maximum displacement = 0.005 Å) with a numeric quality basis set and all‐electron basis with polarization functions. Thermal smearing was applied with a minimum value of ≈0.0005 Ha to promote self‐consistent field convergence and overcome unfavorable electron energy.^[^ ^48^ ^]^ The energies were calculated for the repeating units of the PA complex, PA, and copolymers.

The interaction energies were calculated using Equation (1).

(1)

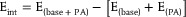

where E_int_ represents interaction energy, E_(base)_ represents the energy of the base copolymer, E_(PA)_ represents the energy of PA, and E_(base + PA)_ represents the total energy of the interacted copolymer with PA.

## Conflict of Interest

The authors declare no conflict of interest.

## Supporting information



Supporting Information

## Data Availability

The data that support the findings of this study are available in the supplementary material of this article.
