# Supplementary material for: Ion‐Pair Interactions in Polyphenylene‐Based Quaternized Membranes Designed for Phosphoric Acid‐Doped Proton Exchange Membranes for High‐Temperature Fuel Cells
Source: Adv Sci (Weinh). 2025 Jul 3;12(37):e09467. doi: 10.1002/advs.202509467 (PMC12499463; doi:10.1002/advs.202509467)
Supplement: Supplementary file 1 — Supporting Information [file ADVS-12-e09467-s001.docx]

**Supporting Information for**

**Ion-Pair Interactions in Polyphenylene-Based Quaternized Membranes Designed for Phosphoric Acid-Doped Proton Exchange Membranes for High-Temperature Fuel Cells**

*Lin Guo, Kenji Miyatake*, Ahmed Mohamed Ahmed Mahmoud, Fanghua Liu, Fang Xian, Vikrant Yadav, Xiaofeng Hao, Shuanjin Wang, Yuezhong Meng*

**EXPERIMENTAL SECTION**

**1. Materials**

Hydrochloric acid (37.0 wt%), bis(1,5-cyclooctadiene)nickel(0) (Ni(cod)_2_), anhydrous toluene, anhydrous dimethylacetamide (DMAc), dimethyl sulfide (DMSO), methanol (MeOH), chloroform, phosphoric acid (85 wt%), sodium chloride (NaCl), sodium hydroxide (NaOH), ethyl acetate, potassium hydroxide (KOH), polyphosphoric acid, sodium carbonate (Na_2_CO_3_), potassium tetrachloroplatinate(II) (K_2_PtCl_4_) and hexane were all purchased from Kanto Chemicals. 2,2’-Bipyridine (BPY), sodium nitrite (NaNO_2_), and copper(I) chloride (CuCl) were purchased from TCI. All chemicals were used as received without purification. AF monomer [6,6'-(2,7-dichloro-9H-fluorene-9,9-diyl)bis(N,N-dimethylhexan-1-amine)] and BAF monomer [(perfluoropropane-2,2-diyl)dibenzene] were synthesized according to our previous report.^[1]^

**2. Synthesis of PBI polymer**

To synthesize the PBI polymer (Scheme S1), polyphosphoric acid (PPA, 80 g), 4,4'-oxydibenzoic acid (1.81 g, 7 mmol), and [1,1'-biphenyl]-3,3',4,4'-tetraamine (1.5 g, 7 mmol) were placed in a 100 mL three-necked round-bottom flask equipped with a stirrer, condenser, and N₂ inlet/outlet. The temperature gradually rose to 150 °C and maintained for 2 h to thoroughly mix the reactants. The reaction mixture was then kept at 190 °C for 10 h. The resulting black viscous mixture was poured into an excess of water, yielding a fibrous product that was filtered and washed multiple times with water. After neutralization with Na₂CO₃ solution, the polymer was washed with water again and dried under vacuum at 80 °C overnight to afford brown solid as 2.73 g (98% yield). The chemical structure of the prepared polymer was confirmed by ^1^H NMR spectra, as shown in Figure S1.


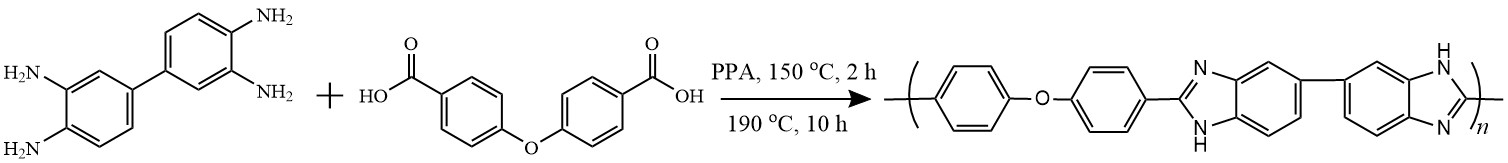


**Scheme S1.** Synthesis of PBI polymer.


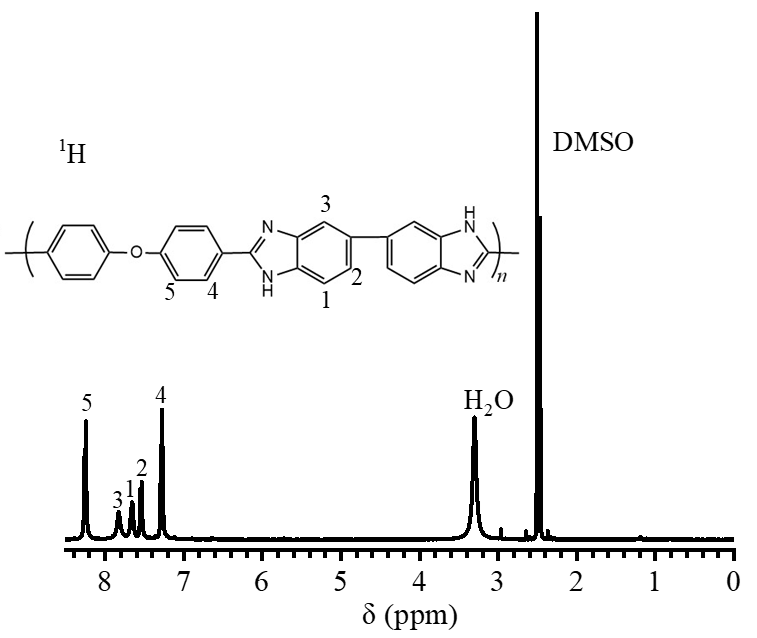


**Figure S1.** ^1^H NMR spectra of prepared PBI polymer in DMSO-*_d6_* at r.t.

**3. Synthesis of 1,1-bis(4-chlorophenyl)cycloheptane (C7) monomer**

Before synthesizing the C7 monomer, 4,4'-(cycloheptane-1,1-diyl)dianiline was synthesized. Excess amount of aniline (30 mL, 330.00 mmol) was added to a solution of cycloheptanone (10.30 g, 91.80 mmol) in 33 mL of 37% hydrochloric acid in a flask. The mixture was stirred at 150 °C for 48 h. After cooling to room temperature, the mixture was made basic with aqueous NaOH to pH ≥ 7. The oil layer was separated, and unreacted aniline was removed by distillation. The residue was further purified by silica gel column chromatography using hexane and ethyl acetate as eluents (hexane: ethyl acetate = 4: 1 and then 1: 1), yielding 4,4'-(cycloheptane-1,1-diyl)dianiline as a pale brown solid (6.44 g, 25% yield).

4,4'-(Cycloheptane-1,1-diyl)dianiline (1.07 g, 3.80 mmol) was dispersed in 4 mL conc. HCl under an ice-water bath, to which a solution of NaNO_2_ (0.62 g, 8.99 mmol) in 5 mL of water was added dropwise while stirring. After 30 minutes, a solution of CuCl (1.10 g, 11.1 mmol) in 6 M HCl (10 mL) cooled to 0 °C was added. The mixture was stirred at room temperature for 1 h, then extracted with chloroform and washed with brine. The organic layer was filtered through a celite plug, washed with water, and evaporated. The obtained crude product was purified by column chromatography using hexane as the eluent. After drying in a vacuum oven and cooling to 5 °C, C7 monomer was obtained as a colorless solid (0.75 g, 62% yield).

**
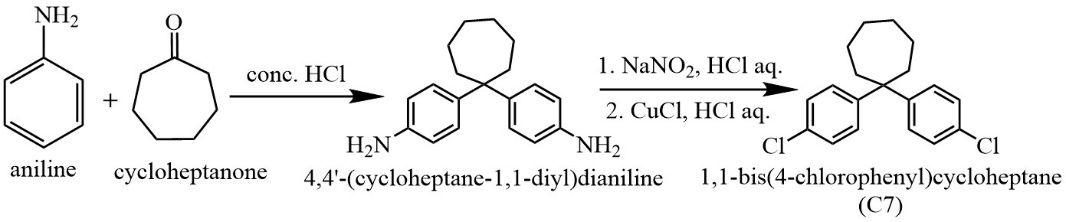
**

**Scheme S2.** Synthesis of monomer C7 monomer.

**
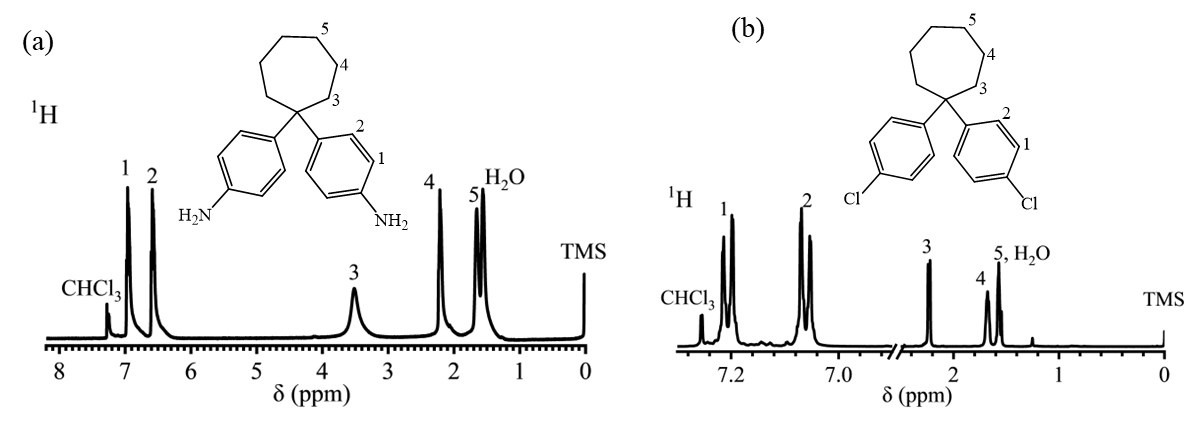
**

**Figure S2.** ^1^H NMR spectra of (a) 4,4'-(cycloheptane-1,1-diyl)dianiline and (b) 1,1-bis(4-chlorophenyl)cycloheptane (C7) monomer in CDCl_3_ at r.t.

**4. Membrane preparation**

The quaternized polymers BAF-QAF and C7-QAF (both in the MeSO₄⁻ form), as well as the as-prepared PBI polymer, were dissolved in DMAc (for BAF-QAF and C7-QAF) or DMSO (for PBI) at a concentration of 5–8% w/v. The resulting solution was then poured onto a flat glass plate and dried at 60 °C for at least 5 h to form flexible, transparent membranes with ca. 50 μm thick. The BAF-QAF and C7-QAF membranes were subsequently immersed in 1 M KOH solution at room temperature for 24 h, followed by immersion in degassed water at room temperature for another 24 h (with the water replaced three times). This process exchanged the MeSO₄⁻ groups in the membranes with OH⁻ groups. The wet membranes were then dried by sandwiching them between weighing papers at room temperature, preparing them for further use.

**5. Phosphoric acid doping of membrane**

Before doping with phosphoric acid (PA), the membrane was dried in a vacuum oven at 60°C overnight. The weight, thickness, length, and width of the membrane were then recorded. The membrane was subsequently immersed in 85 wt% PA at 80 °C for 12 h. After the removal of excess PA from the membrane surface with blotting paper, the PA-doped membrane was assessed again for weight, thickness, length, and width after drying, Figure S2 shows the digital photos of PA-doped membranes. PA uptake and membrane expansion measurements were conducted in quintuplicate, and the final data represents the average values. The following parameters were calculated using specific equations: PA uptake, area swelling ratio, volume swelling ratio, acid doping level (ADL, the number of PA molecules per PA-absorbing site).

$PA uptake= \frac{M_{p}-M_{d}}{M_{d}}\times100\%$ (1)

$Area swelling ratio= \frac{{(A}_{p}-A_{d})}{A_{d}}\times100\%$ (2)

$Volume swelling ratio= \frac{{(V}_{p}-V_{d})}{V_{d}}\times100\%$ (3)

$ADL= \frac{{(M}_{p}-M_{d})\times0.85/M_{PA}}{M_{d}\times IEC}\times100\%$ (4)

where, *M*_p_, *M*_d_ are the mass of the PA-doped membranes and pristine membranes, respectively. *A*_p_, *A*_d_, are the surface area of the PA-doped membranes and pristine membranes, respectively. *V*_p_, and *V*_d_ are the volume size of the PA-doped membranes and pristine membranes, respectively. *M*_PA_ is the molecular weight of phosphoric acid (98 mmol g^-1^) and *IEC* (ion exchange capacity) is the concentration of ammonium group or imidazole group in the polymer backbone (*IEC* is 3.0 mmol g^-1^ for both BAF-QAF and C7-QAF and is 5.0 mmol g^-1^ for PBI in this case).

**6. Measurement and characterization**

**NMR spectra**. ^1^H and ^19^F NMR spectra were measured by a JEOL JNM-ECA ECX500 using deuterated dimethyl sulfoxide (DMSO-*_d_*_6_) or deuterated chloroform (CDCl_3_) containing 1 vol% tetramethylsilane as internal reference.

**Molecular weight**. Molecular weight of the polymers was measured with gel permeation chromatography (GPC) equipped with a Shodex K-805 L column and a Jasco UV 2077 detector (270 nm). CHCl_3_ containing 0.02 M triethylamine was used as eluent and standard polystyrene samples were used for calibration.

**Morphology**. Morphology of the membranes was observed by transmission electron microscopy (TEM, Hitachi H-9500 microscope) with an accelerating voltage of 200 kV. Before the measurement, membrane sample was stained with PtCl_4_^2–^ in 0.5 M K_2_PtCl_4_ aqueous solution, rinsed with pure water, dried in vacuum, embedded in epoxy resin, sectioned to 50 nm thickness, and placed on copper grids. Elemental distribution on the cross-section of the membrane was observed using a scanning electron microscope (SEM, Hitachi SU3500) equipped with an EDS detector (Oxford Instruments) at an accelerating voltage of 15.0 kV.

**Thermogravimetric analysis (TGA).** The thermal stability of the membranes was determined using thermogravimetric analysis. The measurements were performed in N_2_ with a TGA-DSC PERKINELMER STA-8000 at a heating rate of 5 ^o^C min^−1^ from room temperature to 500 °C.

**Tensile test**. Membrane samples were cut into a dumbbell shape (DIN-53503-S3, 35 mm × 6 mm (total) and 12 mm × 2 mm (test area)). The stress versus strain curve was obtained at a stretching rate of 10 mm min^-1^ at 80 ºC with 60% RH and 140 ^o^C with 60% RH in a Shimadzu AGS-J 500N universal testing instrument attached with a Toshin Kogyo Bethel-3A temperature/humidity controllable chamber.

**Molecular weight**. The molecular weight of the polymers was estimated by gel permeation chromatography (GPC) with a UV detector (Jasco 805 UV) and a Shodex K-805L column. DMF containing 0.01 M LiBr was used as the eluent. Measurement was conducted at 50 ºC, and molecular weight was calibrated with standard polystyrene samples.

**Water uptake and proton conductivity**. Water uptake and in-plane proton-conducting resistance (R) of the membranes were measured at 80°C or 120°C using a solid electrolyte analyzer system (MSBAD-V-FC, Bel Japan Co.) in a temperature- and humidity-controllable chamber. Humidity cycling tests (10 cycles were applied for each sample) were carried out with RH ranging from 5% to 90% (at 80°C) or 5% to 40% (at 120°C) with 10% RH interval starting from 10% RH. Each sample was equilibrated for two hours at each RH condition. Before each RH cycle, samples were equilibrated for 8 h at 5% RH. The R value was measured using a four-probe conductivity cell equipped with an AC impedance analyzer (Solartron 1255B and 1287, Solartron Inc.). After equilibrating under a given humidity, the weight of absorbed water in the membrane was automatically measured by a magnetic suspension balance, and simultaneously, the R value was recorded as impedance plots in the frequency range from 1 to 10^5^ Hz. High-temperature resistance (R) of membranes were measured under anhydrous conditions using four probes with a Bio-Logic EC-LAB device equipped with a temperature control system. The temperature increased from 80°C to 160°C in 10°C interval, and each temperature was maintained for 2 hours. Proton conductivity (*σ*) was calculated using the equation: *σ* = L / (A × R), where L is the distance between the two reference electrodes, and A is the cross-sectional area.

***In-situ* fuel cell operation**. Fuel cell evaluation was conducted with a fuel cell test station (YK–S10) at elevated temperature under ambient pressure with dry H_2_ (80 mL min^-1^) and O_2_ (160 mL min^-1^). Before the fuel cell test, MEA was prepared by sandwiching the membrane between two gas diffusion electrodes (2 cm × 2 cm, GDEs) with hot-pressing at 130 ^o^C under the pressure of 2 MPa for 2 min. The GDEs (1 mg cm^−2^ Pt loading, the catalyst contains 40 wt % Pt/C and 15% PTFE as the binder) were obtained from Shanghai Hesen company. The durability test was conducted at a constant current density of 0.15 A cm^−2^ by supplying pure hydrogen (80 mL min^-1^) and air (160 mL min^-1^) to the anode and cathode, respectively.

**Table S1**. The PA uptake, swelling ratio, and acid doping level of membranes after PA-doping.

| Membrane | PA uptake (%) | ADL | Swelling ratio (%) | | Reference |
| --- | --- | --- | --- | --- | --- |
|  |  |  | Area | Volume |  |
| BAF-QAF | 166 | 7.4 | 57 | 129 | This work |
| C7-QAF | 180 | 9.2 | 30 | 66 | This work |
| QPAF-4 | 153 | 7.2 | 59 | 88 | ^[2]^ |
| PBI | 242 | 5.3 | 44 | 192 | This work |
| Quaternized PEEK | ca.160 | N.A. | N.A. | ca.110 | ^[3]^ |
| Quaternized PBI-cross-linked PVC | 236 | 16.1 | N.A. | 134 | ^[4]^ |
| PAEK-PBI | 193 | N.A. | 37 | 79 | ^[5]^ |
| 1-(3-Aminopropyl)imidazole functionalized PVC | 129 | 1.0 | 27 | 65 | ^[6]^ |
| Poly(4-vinylpyridine) | 286 | N.A. | 67 | 164 | ^[7]^ |
| Cross-linked triazole | 175 | 7.5 | 39 | 80 | ^[8]^ |
| Imidazole containing-poly(phenylene oxide) | 323 | 14.1 | 80 | 173 | ^[9]^ |
| Imidazolium silane crosslinked poly(epichlorihydrin)/PTFE | 169 | N.A. | 49 | 119 | ^[10]^ |

**Table S2.** Mechanical properties of membranes assessed at 80 ^o^C with 60% RH and 140 ^o^C with 20% RH.

| Test condition | Sample | Young’s modulus  (GPa) | Yield stress (MPa) | Maximum strain (%) | Rupture energy  (MJ m⁻^3^) |
| --- | --- | --- | --- | --- | --- |
| 80^o^C 60% RH | BAF-QAF | 3.93 | 18.2 | 93 | 17.31 |
| 80^o^C 60% RH | C7-QAF | 2.56 | 15.2 | 79 | 12.36 |
| 80^o^C 60% RH | PA-BAF-QAF | 0.57 | 4.4 | 66 | 3.18 |
| 80^o^C 60% RH | PA-C7-QAF | 0.61 | 4.6 | 43 | 1.96 |
| 80^o^C 60% RH | PA-PBI | 0.35 | 3.8 | 55 | 2.66 |
| 140^o^C 20% RH | BAF-QAF | 3.84 | 15.6 | 24 | 3.47 |
| 140^o^C 20% RH | C7-QAF | 2.45 | 14.3 | 31 | 4.04 |
| 140^o^C 20% RH | PA-BAF-QAF | 0.33 | 2.3 | 83 | 1.79 |
| 140^o^C 20% RH | PA-C7-QAF | 0.96 | 4.9 | 56 | 2.20 |
| 140^o^C 20% RH | PA-PBI | 0.49 | 2.2 | 93 | 3.11 |

**Table S3**. Activation energy of PA-doped membranes calculated from Arrhenius plots.

| Sample | Activation energy (Ea, kJ mol^-1^) | |
| --- | --- | --- |
|  | 80 ^o^C – 120 ^o^C | 120 ^o^C – 160 ^o^C |
| PA-BAF-QAF | 27 | 17 |
| PA-C7-QAF | 25 | 14 |
| PA-PBI | 30 | 19 |

| Membrane | PA uptake (%) | Peak power density  (W cm^-2^) | Temperature (^o^C) | | Reference | |
| --- | --- | --- | --- | --- | --- | --- |
| PA-BAF-QAF | 166 | 0.640 | 160 | This work | |  |
| PA-C7-QAF | 180 | 0.706 | 160 | This work | |  |
| PA-PBI | 242 | 0.442 | 160 | This work | |  |
| Imidazole-rich cross-linked networks | 354 | 0.533 | 160 | ^[11]^ | |  |
| QPAF-4-150%PA | 153 | 0.683 | 160 | ^[2]^ | |  |
| SiO_2_/imidazole-cPBI | 350 | 0.497 | 160 | ^[12]^ | |  |
| Polycation-polybenzimidazole | 220 | 0.680 | 220 | ^[13]^ | |  |
| Sponge-like PBI | 545 | 0.485 | 160 | ^[14]^ | |  |
| Poly(4-vinylpyridine) | 176 | 0.478 | 160 | ^[7]^ | |  |
| Quaternized PBI-cross-linked PVC | 236 | 0.530 | 180 | ^[4]^ | |  |
| Amine-grafted PBI | 374 | 0.409 | 160 | ^[15]^ | |  |
| Free radical scavengers grafted-polyarylethersulfone | 223 | 0.423 | 160 | ^[16]^ | |  |
| poly(ether sulfone benzotriazole) | 221 | 0.427 | 160 | ^[17]^ | |  |
| poly(1-vinylimidazole)-grafted polysulfone | 220 | 0.559 | 160 | ^[18]^ | |  |
| Tetrazole substituted poly-(ether ether  nitrile sulfone) | 112 | 0.287 | 160 | ^[19]^ | |  |
| Methylimidazole-substituted poly(phenylene oxide) | 135 | 0.280 | 160 | ^[20]^ | |  |

**Table S4.** PA uptake and peak power density of PA-doped membranes


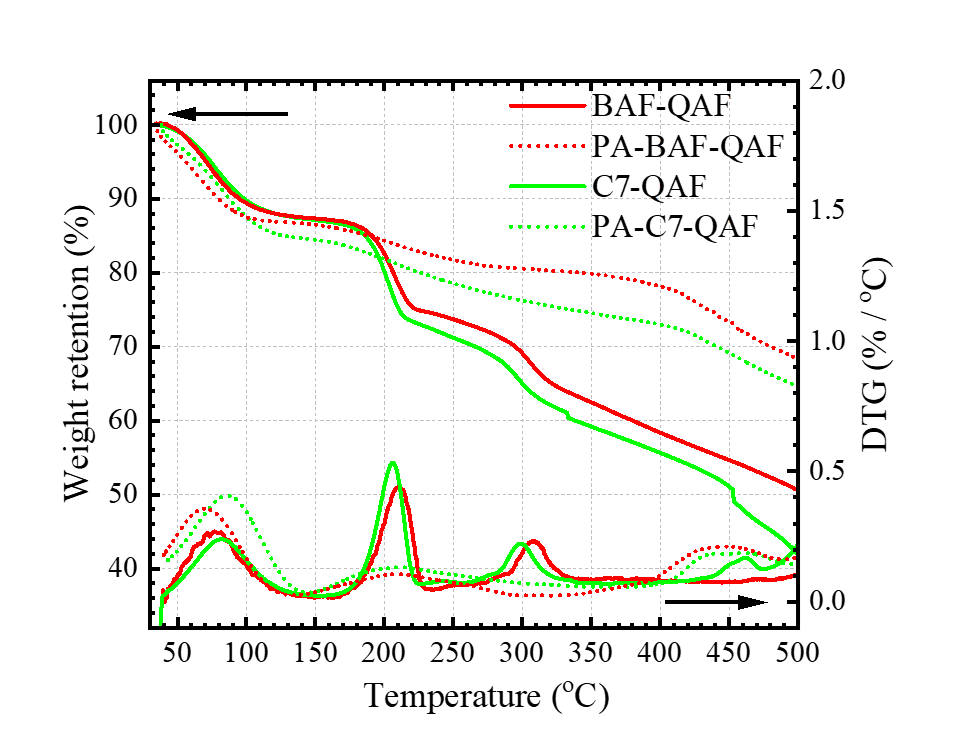


**Figure S3.** TGA curves of BAF-QAF, C7-QAF, PA-BAF-QAF and PA-C7-QAF membranes.


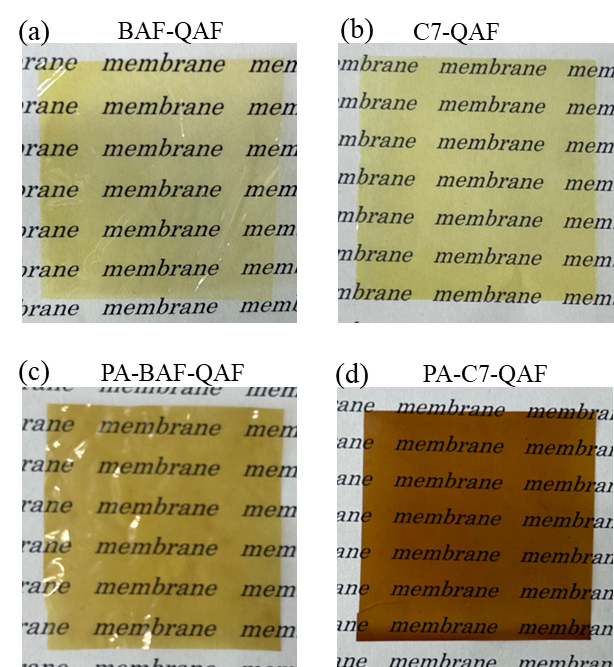


**Figure S4.** Digital photos of (a) BAF-QAF, (b) C7-QAF membranes (both in MeSO_4_^-^ form), and their corresponding membranes (c) PA-BAF-QAF and (d) PA-C7-QAF after PA doping.

**Figure S5.** Chemical shift as a function of acid doping level of PA-C7-QAF, PA-BAF-QAF and PA-PBI membranes.


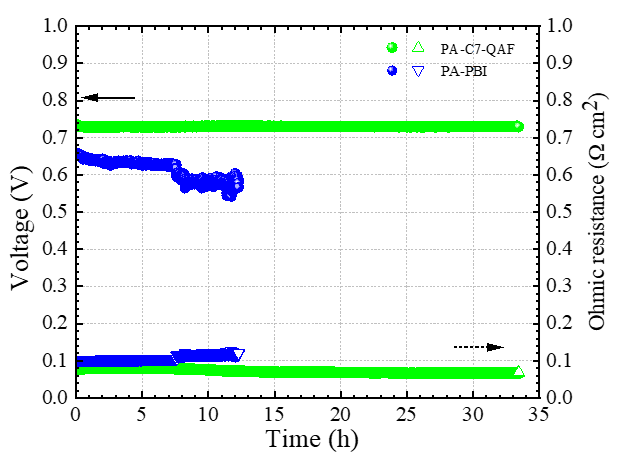


**Figure S6.** Time-dependent cell voltage of PA-C7-QAF and PA-PBI membranes tested under a constant current density of 0.15 A cm⁻² at 140 °C.

**References**

[1] T. Kimura, A. Matsumoto, J. Inukai, K. Miyatake, Highly Anion Conductive Polymers: How Do Hexafluoroisopropylidene Groups Affect Membrane Properties and Alkaline Fuel Cell Performance?, *ACS Appl. Energy Mater.* **2019**, *3*, 469-477.

[2] J. Jiang, Z. Li, M. Xiao, S. Wang, K. Miyatake, Y. Meng, Quaternary ammonium-biphosphate ion-pair based copolymers with continuous H+ transport channels for high-temperature proton exchange membrane, *J. Membr. Sci.* **2022**, *660*, 120878.

[3] N. Zhang, B. Wang, C. Zhao, et al., Quaternized poly (ether ether ketone)s doped with phosphoric acid for high-temperature polymer electrolyte membrane fuel cells, *J. Mater. Chem. A* **2014**, *2*, 13996-14003.

[4] F. Arslan, K. Chuluunbandi, A.T.S. Freiberg, et al., Performance of Quaternized Polybenzimidazole-Cross-Linked Poly(vinylbenzyl chloride) Membranes in HT-PEMFCs, *ACS Appl. Mater. Interfaces* **2021**, *13*, 56584-56596.

[5] J. Jiang, E. Qu, M. Xiao, D. Han, S. Wang, Y. Meng, 3D Network Structural Poly (Aryl Ether Ketone)-Polybenzimidazole Polymer for High-Temperature Proton Exchange Membrane Fuel Cells, *Adv. Polym. Technol.* **2020**, *2020*, 1-13.

[6] R. Liu, Y. Dai, J. Li, X. Chen, C. Pan, J. Yang, Q. Li, 1-(3-Aminopropyl)imidazole functionalized poly(vinyl chloride) for high temperature proton exchange membrane fuel cell applications, *J. Membr. Sci.* **2021**, *620*, 118873.

[7] B. Zhao, D. Ke, Z. Zhang, et al., Poly(4-vinylpyridine) Based Semi-Interpenetrating Cross-Linked High Temperature Proton Exchange Membranes for Fuel Cells, *ACS Appl. Polym. Mater.* **2024**, *6*, 5608-5617.

[8] J. Jang, D.-H. Kim, M.-K. Ahn, et al., Phosphoric acid doped triazole-containing cross-linked polymer electrolytes with enhanced stability for high-temperature proton exchange membrane fuel cells, *J. Membr. Sci.* **2020**, *595*, 117508.

[9] J. Jang, D.H. Kim, B. Kang, J.H. Lee, C. Pak, J.S. Lee, Impact of N-Substituent and pK(a) of Azole Rings on Fuel Cell Performance and Phosphoric Acid Loss, *ACS Appl. Mater. Interfaces* **2021**, *13*, 531-540.

[10] J. Yang, C. Liu, L. Gao, J. Wang, Y. Xu, T. Wang, R. He, Phosphoric acid doped imidazolium silane crosslinked poly(epichlorihydrin)/PTFE as high temperature proton exchange membranes, *RSC Adv.* **2016**, *6*, 61029-61036.

[11] X. Li, H. Ma, P. Wang, et al., Highly Conductive and Mechanically Stable Imidazole-Rich Cross-Linked Networks for High-Temperature Proton Exchange Membrane Fuel Cells, *Chem. Mat.* **2020**, *32*, 1182-1191.

[12] X. Li, H. Ma, P. Wang, et al., Construction of High-Performance, High-Temperature Proton Exchange Membranes through Incorporating SiO(2) Nanoparticles into Novel Cross-linked Polybenzimidazole Networks, *ACS Appl. Mater. Interfaces* **2019**, *11*, 30735-30746.

[13] G. Venugopalan, K. Chang, J. Nijoka, S. Livingston, G.M. Geise, C.G. Arges, Stable and Highly Conductive Polycation–Polybenzimidazole Membrane Blends for Intermediate Temperature Polymer Electrolyte Membrane Fuel Cells, *ACS Appl. Energy Mater.* **2019**, *3*, 573-585.

[14] K. Geng, H. Tang, Q. Ju, H. Qian, N. Li, Symmetric sponge-like porous polybenzimidazole membrane for high temperature proton exchange membrane fuel cells, *J. Membr. Sci.* **2021**, *620*, 118981.

[15] G. Liu, H. Pan, S. Zhao, Y. Wang, H. Tang, H. Zhang, Grafting of Amine End-Functionalized Side-Chain Polybenzimidazole Acid-Base Membrane with Enhanced Phosphoric Acid Retention Ability for High-Temperature Proton Exchange Membrane Fuel Cells, *Molecules* **2024**, *29*, 340.

[16] J. Wang, Y. Dai, R. Wan, W. Wei, S. Xu, F. Zhai, R. He, Grafting free radical scavengers onto polyarylethersulfone backbones for superior chemical stability of high temperature polymer membrane electrolytes, *Chem. Eng. J.* **2021**, *413*, 127541.

[17] K. Wang, L. Yang, W. Wei, L. Zhang, G. Chang, Phosphoric acid-doped poly(ether sulfone benzotriazole) for high-temperature proton exchange membrane fuel cell applications, *J. Membr. Sci.* **2018**, *549*, 23-27.

[18] H. Bai, H. Wang, J. Zhang, J. Zhang, S. Lu, Y. Xiang, High temperature polymer electrolyte membrane achieved by grafting poly(1-vinylimidazole) on polysulfone for fuel cells application, *J. Membr. Sci.* **2019**, *592*, 117395.

[19] D. Henkensmeier, N.M.H. Duong, M. Brela, et al., Tetrazole substituted polymers for high temperature polymer electrolyte fuel cells, *J. Mater. Chem. A* **2015**, *3*, 14389-14400.

[20] W. Wu, G. Zou, X. Fang, C. Cong, Q. Zhou, Effect of Methylimidazole Groups on the Performance of Poly(phenylene oxide) Based Membrane for High-Temperature Proton Exchange Membrane Fuel Cells, *Ind. Eng. Chem. Res.* **2017**, *56*, 10227-10234.
